# Effects of anthropogenic stress on hosts and their microbiomes: Treated wastewater alters performance and gut microbiome of a key detritivore (*Asellus aquaticus*)

**DOI:** 10.1111/eva.13540

**Published:** 2023-03-30

**Authors:** Elvira Lafuente, Louis Carles, Jean‐Claude Walser, Marco Giulio, Simon Wullschleger, Christian Stamm, Katja Räsänen

**Affiliations:** ^1^ Eawag: Swiss Federal Institute of Aquatic Science and Technology Dübendorf Switzerland; ^2^ Instituto Gulbenkian de Ciência Oeiras Portugal; ^3^ Department of Environmental Systems Science D‐USYS, Genetic Diversity Centre Swiss Federal Institute of Technology (ETH), Zürich Zürich Switzerland; ^4^ Department of Biological and Environmental Science University of Jyväskylä Jyväskylä Finland

**Keywords:** chemical pollution, environmental stress, freshwater ecosystems, host–microbiome interactions, isopods

## Abstract

Human activity is a major driver of ecological and evolutionary change in wild populations and can have diverse effects on eukaryotic organisms as well as on environmental and host‐associated microbial communities. Although host–microbiome interactions can be a major determinant of host fitness, few studies consider the joint responses of hosts and their microbiomes to anthropogenic changes. In freshwater ecosystems, wastewater is a widespread anthropogenic stressor that represents a multifarious environmental perturbation. Here, we experimentally tested the impact of treated wastewater on a keystone host (the freshwater isopod *Asellus aquaticus*) and its gut microbiome. We used a semi‐natural flume experiment, in combination with 16S rRNA amplicon sequencing, to assess how different concentrations (0%, 30%, and 80%) of nonfiltered wastewater (i.e. with chemical toxicants, nutrients, organic particles, and microbes) versus ultrafiltered wastewater (i.e. only dissolved pollutants and nutrients) affected host survival, growth, and food consumption as well as mid‐ and hindgut bacterial community composition and diversity. Our results show that while host survival was not affected by the treatments, host growth increased and host feeding rate decreased with nonfiltered wastewater – potentially indicating that *A. aquaticus* fed on organic matter and microbes available in nonfiltered wastewater. Furthermore, even though the midgut microbiome (diversity and composition) was not affected by any of our treatments, nonfiltered wastewater influenced bacterial composition (but not diversity) in the hindgut. Ultrafiltered wastewater, on the other hand, affected both community composition and bacterial diversity in the hindgut, an effect that in our system differed between sexes. While the functional consequences of microbiome changes and their sex specificity are yet to be tested, our results indicate that different components of multifactorial stressors (i.e. different constituents of wastewater) can affect hosts and their microbiome in distinct (even opposing) manners and have a substantial impact on eco‐evolutionary responses to anthropogenic stressors.

## 1 INTRODUCTION

Human activities have dramatically altered global and local ecosystems and act as strong selective agents in natural populations (e.g. Hoffmann & Parsons, 1997; Alberti, 2015). The effects of such anthropogenic activities in nature scale from genes and individuals, and from eukaryotes and microbes, to entire ecosystems (Cavicciolli et al., 2019; Maltby, 1999). Yet, the influence of anthropogenic change on host–microbiome interactions is still poorly understood – despite their potential relevance for host performance and evolution (Koskella et al., 2017). This is, at least partly, because most environmental and ecological studies have focused on understanding how stressors, such as chemical pollution (Backhaus et al., 2012; Chonova et al., 2018; Yang et al., 2000), affect species diversity, while most evolutionary ecological studies have focused on the contribution of genes and/or phenotypic plasticity to shaping organismal responses to stress (Gienapp et al., 2008; Matesanz et al., 2010; Merilä & Hendry, 2014; Rodrigues & Beldade, 2020; Taddei et al., 1997). Thus, awareness of the role of the organism's microbiome (i.e. communities of microorganisms associated with a host) as an important player in driving the evolution of its hosts (Foster et al., 2017; Henry et al., 2021; Koskella et al., 2017), has only recently emerged.

The gut microbiome is of particular relevance in relation to organismal responses to stress (Kinross et al., 2011; Shreiner et al., 2015). Gut‐associated bacteria can, for instance, respond rapidly to changes in the host environment and influence host fitness (e.g. Voolstra & Ziegler, 2020) by altering host metabolism (e.g. Shin et al., 2011), morphology (e.g. Tapia et al., 2016), and immunity (e.g. Thaiss et al., 2016). Ecotoxicological studies further indicate that gut microbes can degrade and metabolize toxic compounds and thus modulate the toxicity of certain chemicals to the host (Ceja‐Navarro et al., 2015; Daisley et al., 2018; Itoh et al., 2018). Importantly, from an eco‐evolutionary perspective, the gut microbiome can influence host adaptive trajectories by expanding host dietary niches (e.g. Aizpurua et al., 2021) and by influencing host responses to environmental change (e.g. Avila‐Magaña et al., 2021). It has even been proposed that the microbiome can be a source of phenotypic plasticity, whereby individuals faced with novel environmental challenges can adapt through changes in their microbiome (Koskella et al., 2017). Yet, empirical assessments of the extent to which the microbiome mediates host adaptation under stressful conditions remain scarce, notably in the context of stressors arising from human activities.

Many of the current threats to wildlife and natural ecosystems consist of multifactorial stressors, that is a combination of several abiotic and/or biotic stress factors that simultaneously affect an organism or a population (e.g. Zandalinas et al., 2021). While the effects of isolated stressors have been thoroughly investigated in many ecological and evolutionary studies on individuals (e.g. Fischer et al., 2004), populations (Frago & Bauce, 2014; Krebs & Loeschcke, 1995), and ecosystems (e.g. García et al., 2018), research on multifactorial stressors is less common. However, natural systems comprise complex environments which include variation in multiple and highly dynamic environmental cues, that can interact in different manners (e.g. additively, synergistically, or antagonistically; Crain et al., 2008), mask each other's effects (e.g. Burdon et al., 2020), and, therefore, impact organismal phenotype and population persistence in diverse ways (Breitburg et al., 1998; Crain et al., 2008; Piggott et al., 2015; Singh et al., 2020; Vinebrooke, 2004). In this context, the additional complexity arising from host responses to complex stressors depending on host‐associated microbes (not only host physiology or genotype; e.g. Henry et al., 2019, 2021; Koskella et al., 2017) can complicate the predictions about responses of natural populations to anthropogenic change. Moreover, the consequences of host–microbiome interactions can be sex‐specific (Bates et al., 2022; Bolnick et al., 2014), an aspect that is thus far little studied in natural populations.

In aquatic ecosystems, treated wastewater is a ubiquitous multifactorial stressor of anthropogenic origin (Breitburg et al., 1998; Piggott et al., 2015; Sala et al., 2000; Schwarzenbach et al., 2006). Wastewater is a complex mix of nutrients, chemical contaminants, microbes, and organic matter (Stamm et al., 2016), and its discharge to the receiving environment (in the form of treated wastewater from wastewater treatment plants) leads to increased loads of nutrients, chemical pollution (e.g. Reid et al., 2019), microbes (e.g. Mansfeldt et al., 2020), and organic matter (e.g. Petrie et al., 2015), as well as to altered temperatures (e.g. Stamm et al., 2016). Although modern wastewater treatment plants are usually effective in removing nutrients, the input of microbes and a highly complex mix of micropollutants (i.e. low concentration pharmaceuticals, pesticides, and industrial chemicals) can be substantial (Stamm et al., 2016) and negatively influence aquatic organisms as well as ecosystems (Eggen et al., 2014; Schwarzenbach et al., 2006). In particular, wastewater can alter both macroinvertebrate (Aristone et al., 2022; Burdon et al., 2019) and microbial communities (Carles et al., 2021; Chonova et al., 2018; Price et al., 2018; Tamminen et al., 2021), with pollution‐tolerant taxa and pathogenic and antibiotic‐resistant microbes being reported in polluted areas (Burdon et al., 2020; Carles et al., 2021; Tlili et al., 2017).

While chemical contaminants can be strong selective factors in natural populations (Loria et al., 2019; Palumbi, 2009), the combined effects of chemical pollution in presence of other stressors on host–microbiome responses are poorly understood. Given the complexity and pervasiveness of wastewater, studies on effects of wastewater constituents provide an interesting opportunity for understanding both host and host‐associated microbiome responses to multifactorial stressors. Importantly, chemical toxicants, nutrients, and microbes have the potential to affect both the host and its microbiome via effects on host physiology and resource availability or via effects on environmental microbes (Adamovsky et al., 2018; Konschak et al., 2020). Ultimately, the effects of wastewater on organismal‐level responses may affect ecological and evolutionary processes, and even drive eco‐evolutionary feedbacks, especially when keystone species are affected (Alberti, 2015; Hendry et al., 2017). To date, however, only a few studies have integrated ecological, ecotoxicological, and evolutionary ecology (Gessner & Tlili, 2016) in order to understand host–microbiome responses to human stressors.

We studied the impact of treated wastewater on a sexually dimorphic detritivore host (the freshwater isopod *Asellus aquaticus*) and its gut microbiome and explored to what extent responses differ between males and females. *Asellus aquaticus* is a particularly well‐suited and ecologically relevant species for such integrative studies (Lafuente et al., 2021), as it is a keystone species with a broad environmental tolerance (including resilience to high levels of pollution; Maltby, 1991, 1995) that feeds both on microbes and decaying plant matter (Graça et al., 1993; Kemp et al., 2020; Lafuente et al., 2021). Importantly, *A. aquaticus* (as many other isopod species) has a highly diverse gut microbiota in its hepatopancreas (i.e. midgut gland, thereafter called “midgut”) and hindgut (Bredon et al., 2020), with important eco‐evolutionary roles (Bouchon et al., 2016; Liao et al., 2023). Hepatopancreatic symbionts, for example, are thought to enable isopods to digest leaf litter as well as to aid the host in detoxifying chemical compounds (Bouchon et al., 2016; Bredon et al., 2020; Zimmer & Bartholmé, 2003).

To investigate the effects of wastewater on host–microbiome interactions, we used a semi‐natural experiment, combined with 16S rRNA amplicon sequencing of the host mid‐ and hindgut microbiomes. We manipulated the amount (concentration) and composition (i.e. with or without ultrafiltration) of conventionally treated wastewater to test how nonfiltered wastewater (i.e. including microbes, micropollutants, particulate matter, and nutrients) *versus* ultra‐filtered wastewater (i.e. containing only dissolved chemicals, including micropollutants and nutrients) affected host performance (survival, growth, and food consumption) and the diversity and composition of mid‐ and hindgut bacterial communities. We explicitly compared males and females to assess degree of sexual dimorphism in host microbiome responses.

We predicted that negative effects of chemical pollutants in wastewater would manifest as changes in growth and/or feeding rates across treatments, and lead to changes in community composition of gut‐associated bacteria. In particular, we expected to see the effects of chemical pollution as a difference between nonfiltered and ultrafiltered (i.e. when microbes and organic particles have been removed) wastewater. This effect could arise either if other components of the nonfiltered wastewater would mask negative effects of chemical pollutants (e.g. Burdon et al., 2020) or provide different (and more) sources of environmental bacteria and dietary sources (i.e. organic particles; Graça et al., 1993). Exploring these different scenarios provides insight into how a multifactorial and pervasive anthropogenic stressor can act as a selective force in natural populations impacting host–microbiome interactions.

## 2 MATERIALS AND METHODS

### 2.1 Study system

All individuals used in this study belong to the species *Asellus aquaticus* and were sampled in Lake Lucerne at Kastanienbaum, Horw (Switzerland; coordinates: 47°00′07.6″N 8°20′01.3″E). This sampling site is relatively unimpacted by chemical pollution, and there are no wastewater treatment plants in proximity to the sampling site (Götz, 2013). The *A. aquaticus* population at this sampling site has a high year‐around density, is phenotypically variable, and has been successfully used in mesocosm and laboratory experiments (Lürig et al., 2019, 2021).

Animals were collected from uprooting macrophytes (mainly of *Chara tomentosa*) in shallow water with kick nets and subsequent flushing of the vegetation. Upon collection, animals were transported in containers containing freshwater to the Swiss Federal Institute of Aquatic Science (Eawag) in Dübendorf (Switzerland). Until the start of the experiment, all animals were kept under standardized laboratory conditions in 4.5‐L tanks, at a density of 60–90 animals per tank, in a flow‐through system with animal‐proof water (e.g. without elevated concentrations of metals). In the system, water was replaced at approximately 400 mL every 24 h, kept at an average temperature of 18°C, with a light–dark cycle of 16:8 h. The animals were fed *ad libitum* with conditioned leaves (i.e. soaked in animal‐proof water for at least 2 weeks) of black alder (*Alnus glutinosa*).

### 2.2 Experimental design

#### 2.2.1 Experimental system

The experiment was conducted in an artificial flume system, called Maiandros, that allows experimental manipulation of the proportion of wastewater (or dosing of different chemicals) in a replicated manner (Burdon et al., 2020; Carles et al., 2021). The Maiandros was located in an experimental hall and fed by wastewater from the wastewater treatment plant at Eawag.

**FIGURE 1:**
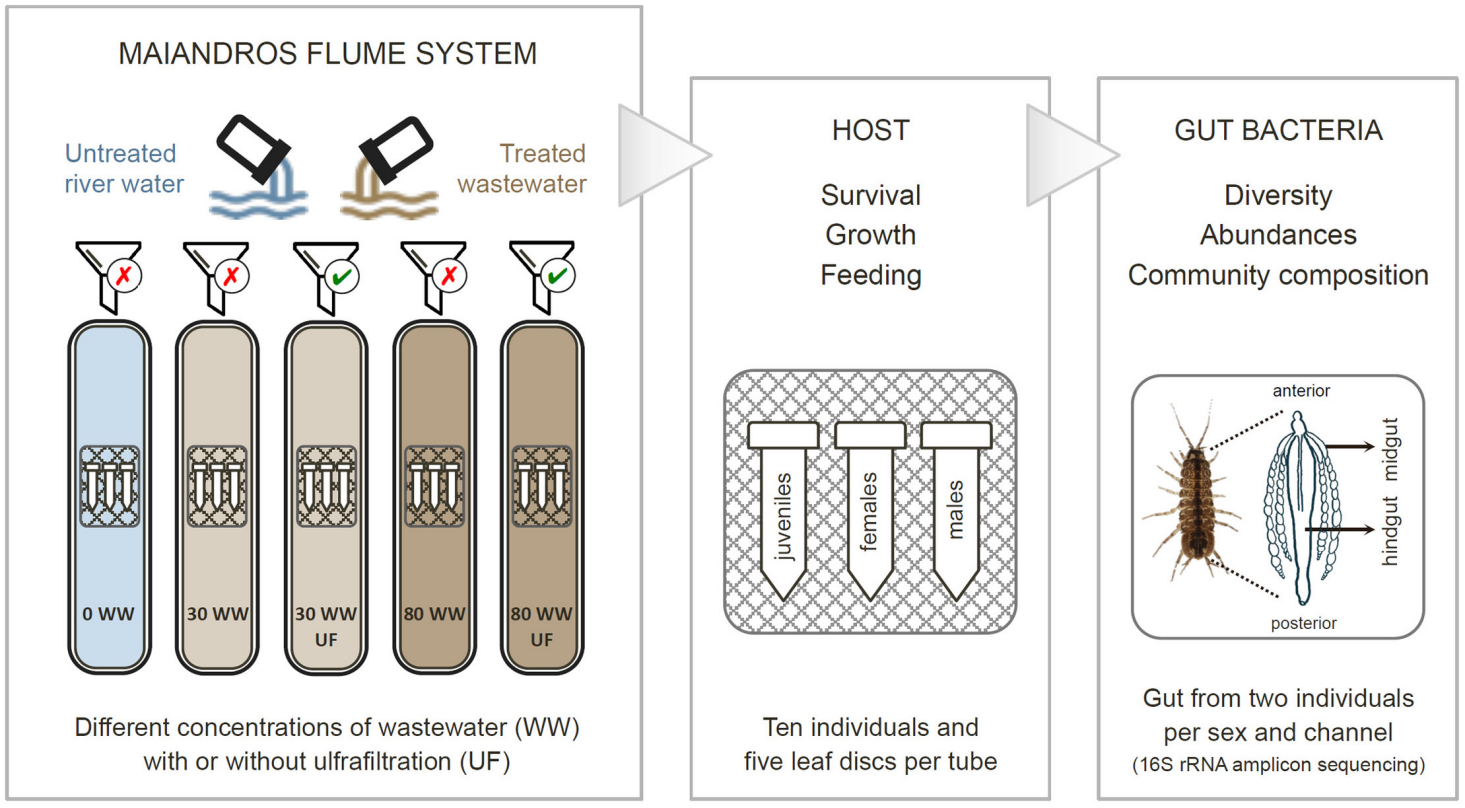
Experimental design. There were five treatments assigned randomly across 20 channels: river water only (without wastewater or ultrafiltration; 0% WW) and two concentrations of wastewater (30%WW and 80%WW) with ultrafiltration (30% WW‐UF and 80% WW‐UF) or without ultrafiltration (30% WW and 80% WW). Within each channel, there were three experimental containers, each containing 10 individuals (juveniles, males, or females) and five leaf discs. Host performance was measured as survival, growth, and food consumed. For analyses of the gut microbiome, a subset of two males and two females (per experimental container) were dissected to collect midguts and hindguts, which were used for 16S rRNA amplicon sequencing.

In the current study, we manipulated wastewater (WW) concentration (i.e. dilution) and composition (i.e. using ultrafiltration, UF) to test the effects of wastewater *versus* dissolved chemicals on *A. aquaticus* and its microbiome (Figure 1). Ultrafiltration removes wastewater‐associated microbes and organic particles, reflecting water chemistry. The experiment consisted of five treatments distributed across 20 flumes in the Maiandros: untreated river water (0% WW; from Chriesbach river just outside the experimental hall) or river water mixed with two concentrations of wastewater (30% WW and 80% WW) and with ultrafiltration (30% WW UF and 80% WW UF) or without ultrafiltration (30% WW non‐UF and 80% WW non‐UF) (Figure 1). The experimental treatments were assigned randomly across the 20 flumes and configured in five blocks, with four replicate channels per treatment. The dose of each treatment (i.e. flow of wastewater) into the flumes was continuous at a rate of 80 L/h per channel for the entire duration of the experiment. The ultrafiltration unit was described by (Desiante et al., 2022). Briefly, the system consists in a set of membranes with a nominal pore size of 0.4 μm, allowing for the removal of particulates, including microorganisms, from the effluent. Channel dimensions had a length of 2.6 m, a width of 0.15 m, and a water depth of 0.1 m, and the permeate flow was maintained at 320 L/h during the experiment. Due to the partial recirculation, the water had a mean residence time in the flumes of about 10 min.

#### 2.2.2 Animals

The experimental design for *A. aquaticus* consisted of 10 individuals for each of three classes: males, females, and juveniles. The animals were housed in experimental containers (see below) and distributed randomly among the 20 channels and the five treatments. Thus, there were three experimental containers per channel and a total of 40 individuals for each class per treatment (Figure 1). Individuals were assigned to adults and juveniles based on body size, with individuals <4 mm in length being juveniles (sexual maturity in *A. aquaticus* happens at approximately 4 mm; e.g. Marcus et al., 1978). Sexing of adults was done by examining the pleopods (Kemp et al., 2020). At the beginning and the end of the experiment, all animals were placed individually in 6‐well plates (sterile polystyrene BIOFIL culture plates) and photographed with a digital camera (Canon EOS 750D camera, with a Canon EFS 18‐55 mm Image Stabilizer MACRO 0.25 m/0.8 ft lens) for later measurements of body size.

The experimental containers with animals and leaf discs were introduced in the Maiandros 10 days after the system had been set up. The experiment ran for 14 days, between 20/11/2019 and 04/12/2019. These experimental containers consisted of modified 50 mL falcon tubes, where the lid and the bottom of the tube were cut open and covered with a mesh (0.5 mm mesh size) to allow water flow (Figure 1). Three falcon tubes were attached to a ceramic tile structure, and the three‐tube set was then placed inside each experimental channel (Figure 1). Each experimental container also contained five standard‐sized (18 mm diameter) leaf discs of black alder (*A. glutinosa*) as a food source. The leaf discs had been previously soaked in animal‐proof water for two weeks to facilitate microbial growth and *A. aquaticus* feeding (Graca et al., 1993). The leaf discs were photographed at the start and the end of the experiment by placing multiple leaf discs on transparent film prior to imaging.

### 2.3 Measured background, host, and host–microbiota response variables

To assess the efficacy and the effects of the experimental manipulations on environmental conditions experienced by *A. aquaticus*, we measured the following background variables: ultrafiltration efficiency (measured via quantification of microbial loading) and water physicochemistry (nutrient, micropollutant, and metal concentrations), as described below.

#### 2.3.1 Background variables

##### Wastewater ultrafiltration efficiency

Ultrafiltration efficiency was assessed prior to introducing *A. aquaticus* into the system by measuring the bacterial abundance in the wastewater buffer tank and directly after ultrafiltration of the wastewater (Table S1). Bacterial abundance was estimated by flow cytometry according to (Carles et al., 2021) with a few modifications. Briefly, 5 mL from each wastewater sample was added to 5 mL of phosphate‐buffered formalin (2% formaldehyde, 0.2% sodium pyrophosphate, final concentrations) and stored at 4°C until analysis. After an ultrasonic treatment for 3 × 20 s (Branson Digital Sonifier 250), the samples were diluted, stained with SYBR® Green I (1 × final concentration; Promega), and incubated at room temperature in the dark for 15 min. Fluorescent beads (Flow‐count fluorospheres; Beckman Coulter) with a known concentration were spiked to the samples as a standard to determine the cell abundance. The samples were analyzed using a Gallios flow cytometer (Beckman Coulter). Cell counts were used to assess the percentage of bacteria that was removed by ultrafiltration.

##### Water physico‐chemistry

Abiotic parameters (pH, temperature, conductivity, and oxygen concentration) were measured daily directly from all 20 channels using a multi‐parameter portable meter (WTW Meters) (Tables 1, S2). Additionally, water samples were taken every week in two replicate channels per treatment (i.e. 10 of the 20 channels) for the measurement of 16 water quality parameters (Tables 1, S3). Water quality parameters (mostly nutrients) were measured using standard methods, as described by the Swiss National River Monitoring and Survey Programme (FOEN, 2020).

**TABLE 1:** Water parameters among treatments.

Type of parameter	Parameter	Treatment	0_WW	30_WW	30UF_WW	80_WW	80UF_WW
		WW (%)	0	30	30	80	80
		Ultrafiltration	Non‐UF	Non‐UF	UF	Non‐UF	UF
Abiotic parameter							
	Conductivity		794.72 ± 78.53	993 ± 103.73	974.57 ± 101.64	1358.5 ± 200.21	1255.63 ± 148.85
	O_2_		10 ± 0.4	8.92 ± 0.46	9.91 ± 0.37	7.09 ± 0.66	9.75 ± 0.43
	pH		8.13 ± 0.14	7.95 ± 0.19	8.23 ± 0.1	7.72 ± 0.09	8.38 ± 0.08
	Temperature		13.71 ± 0.61	14.31 ± 0.49	13.87 ± 0.56	15.17 ± 0.4	14.09 ± 0.57
Water quality							
	Ammonium (μg L^−1^)		9.9 ± 2.51	22.42 ± 10.03	13.58 ± 4.88	85 ± 16.96	21.07 ± 9.66
	Calcium (mg L^−1^)		111.12 ± 1	113.03 ± 5.56	113.37 ± 3.24	121.89 ± 13.66	115.51 ± 8.59
	Chloride (mg L^−1^)		50.52 ± 6.51	96.45 ± 22.62	94.24 ± 15.47	210.02 ± 61.41	167.95 ± 39.48
	Dissolved Organic Carbon (mg L^−1^)		2.04 ± 0.14	3.27 ± 0.52	3.16 ± 0.19	6.06 ± 1.36	5.05 ± 0.17
	Magnesium (mg L^−1^)		18.13 ± 0.56	19.25 ± 1.43	19.21 ± 1.26	22.51 ± 3.21	21.02 ± 2.36
	Natrium (mg L^−1^)		32.15 ± 4.7	66.44 ± 13.27	65.89 ± 6.98	140.98 ± 30.88	120.86 ± 19.77
	Nitrate (mg L^−1^)		6.01 ± 0.18	6.41 ± 0.27	6.48 ± 0.15	6.6 ± 0.52	7.26 ± 0.56
	Nitrite (μg L^−1^)		7.36 ± 0.45	34.15 ± 14.29	28.53 ± 6.56	113.75 ± 32.46	67.25 ± 19.74
	Phosphate (μg L^−1^)		100.06 ± 15.92	212.1 ± 165.04	162.98 ± 80.38	230.07 ± 232.24	254.07 ± 221.59
	Potassium (mg L^−1^)		4.3 ± 0.6	6.44 ± 0.79	6.13 ± 0.35	10.16 ± 0.66	9.87 ± 0.63
	Silicic acid (mg L^−1^)		15.3 ± 0.51	15.73 ± 2.46	16.77 ± 0.58	20 ± 0.81	19.48 ± 1.44
	Sulfate (mg L^−1^)		28.16 ± 2.57	31.35 ± 2.11	31.38 ± 2.01	37.33 ± 1.4	37.08 ± 2.26
	Total Inorganic Carbon (mg L^−1^)		75.56 ± 1.36	80.07 ± 2.9	80.17 ± 2.12	89.8 ± 5.6	87.93 ± 5.58
	Total Nitrogen (mg L^−1^)		6.24 ± 0.11	6.79 ± 0.31	6.72 ± 0.23	7.83 ± 1.1	7.59 ± 0.63
	Total Organic Carbon (mg L^−1^)		2.3 ± 0.25	4.03 ± 0.62	3.36 ± 0.16	11.49 ± 4.09	5.42 ± 0.17
	Total Phosphorus (μg L^−1^)		115.43 ± 20.72	425.25 ± 174.55	254.25 ± 89.39	1393.25 ± 662.42	444.58 ± 245.91
Micropollutants		Substance group					
	Acesulfame	Artificial sweetener	2 ± 1	8 ± 4	8.33 ± 4.93	10 ± 3	11.67 ± 3.06
	Cyclamate	Artificial sweetener	<LOQ	<LOQ	1.33 ± 0.58	<LOQ	1.67 ± 1.15
	4/5‐Methylbenzotriazole	Corrosion inhibitor	2 ± 1	7.67 ± 3.06	6 ± 2	12 ± 3	12.33 ± 2.08
	Benzotriazole	Corrosion inhibitor	2 ± 1	7.33 ± 1.53	5.67 ± 2.08	12.67 ± 1.53	12.33 ± 2.52
	2,6‐Dichlorbenzamide	Pesticide	<LOQ	<LOQ	<LOQ	<LOQ	<LOQ
	Carbendazim (Azole)	Pesticide	<LOQ	<LOQ	<LOQ	<LOQ	<LOQ
	Chloridazone‐methyl‐desphenyl	Pesticide	13 ± 2.65	7.33 ± 4.04	10.67 ± 3.21	4 ± 2.65	5 ± 3.61
	Chlortoluron	Pesticide	<LOQ	<LOQ	<LOQ	<LOQ	<LOQ
	DEET	Pesticide	<LOQ	3 ± 1	4 ± 2.65	9 ± 2	10 ± 2
	Diazinon	Pesticide	<LOQ	<LOQ	<LOQ	<LOQ	<LOQ
	Dimethenamid	Pesticide	<LOQ	<LOQ	<LOQ	<LOQ	<LOQ
	Dimethoate	Pesticide	<LOQ	<LOQ	<LOQ	<LOQ	<LOQ
	Diuron	Pesticide	<LOQ	<LOQ	<LOQ	<LOQ	<LOQ
	Epoxiconazole	Pesticide	<LOQ	<LOQ	<LOQ	<LOQ	<LOQ
	Ethofumesate	Pesticide	<LOQ	<LOQ	<LOQ	<LOQ	<LOQ
	Fipronil	Pesticide	<LOQ	<LOQ	<LOQ	<LOQ	<LOQ
	Isoproturon	Pesticide	<LOQ	<LOQ	<LOQ	<LOQ	<LOQ
	Mecoprop	Pesticide	<LOQ	<LOQ	<LOQ	<LOQ	<LOQ
	Metamitron	Pesticide	<LOQ	<LOQ	<LOQ	<LOQ	<LOQ
	Metamitron‐Desamino‐4	Pesticide	<LOQ	<LOQ	<LOQ	<LOQ	<LOQ
	Metolachlor‐OXA	Pesticide	<LOQ	<LOQ	<LOQ	<LOQ	<LOQ
	Pirimicarb	Pesticide	<LOQ	<LOQ	<LOQ	<LOQ	<LOQ
	Propiconazole	Pesticide	<LOQ	<LOQ	<LOQ	<LOQ	<LOQ
	Tebuconazole	Pesticide	<LOQ	<LOQ	<LOQ	<LOQ	<LOQ
	Terbutryn	Pesticide	<LOQ	<LOQ	<LOQ	<LOQ	<LOQ
	4‐Acetamidoantipyrine	Pharmaceutical	<LOQ	4.33 ± 1.53	4.67 ± 2.52	10.33 ± 2.08	10.67 ± 2.08
	4‐Formylaminoantipyrine	Pharmaceutical	<LOQ	5 ± 2	4 ± 2	10.67 ± 2.08	10.33 ± 2.08
	Amisulpride	Pharmaceutical	<LOQ	5.33 ± 2.08	3.67 ± 1.53	10.67 ± 2.52	10.33 ± 1.53
	Atenolol	Pharmaceutical	<LOQ	4.33 ± 1.53	4.67 ± 2.52	10.67 ± 1.53	10.33 ± 2.52
	Candesartan	Pharmaceutical	1.33 ± 0.58	6 ± 2	5 ± 2	11.67 ± 2.08	11.33 ± 2.08
	Carbamazepine	Pharmaceutical	<LOQ	4.67 ± 2.08	4.33 ± 2.08	10.33 ± 2.52	10.67 ± 1.53
	Cetirizine	Pharmaceutical	<LOQ	<LOQ	<LOQ	4.67 ± 1.53	4.33 ± 2.52
	Clarithromycin	Pharmaceutical	<LOQ	4.67 ± 1.53	4.33 ± 2.52	11.33 ± 1.53	9.67 ± 2.08
	Clopidogrel Carboxylic Acid	Pharmaceutical	<LOQ	5.33 ± 2.08	3.67 ± 1.53	10.67 ± 2.08	10.33 ± 2.08
	Diclofenac	Pharmaceutical	<LOQ	5 ± 2	4 ± 2	11 ± 2.65	10 ± 1
	Gabapentin	Pharmaceutical	2 ± 1	7 ± 1	6 ± 2.65	12.67 ± 1.53	12.33 ± 2.52
	Hydrochlorothiazide	Pharmaceutical	<LOQ	5.33 ± 2.08	3.67 ± 1.53	9.67 ± 2.08	11.33 ± 1.53
	Lamotrigine	Pharmaceutical	2 ± 1	7 ± 2	6 ± 2	12.67 ± 2.08	12.33 ± 2.08
	Levetiracetam (fragment)	Pharmaceutical	<LOQ	<LOQ	<LOQ	1.67 ± 1.15	1.33 ± 0.58
	Lidocaine (Diocaine)	Pharmaceutical	<LOQ	4.67 ± 2.08	4.33 ± 2.08	10.67 ± 1.53	10.33 ± 2.52
	Mefenamic acid	Pharmaceutical	<LOQ	4.67 ± 1.53	4.33 ± 2.52	10.67 ± 1.53	10.33 ± 2.52
	Metoprolol	Pharmaceutical	<LOQ	5 ± 2	4 ± 2	10.67 ± 2.08	10.33 ± 2.08
	Naproxen	Pharmaceutical	<LOQ	<LOQ	<LOQ	5 ± 2	4 ± 2
	Oxazepam	Pharmaceutical	<LOQ	<LOQ	<LOQ	4.67 ± 2.08	4.33 ± 2.08
	Sitagliptin	Pharmaceutical	2 ± 1	7.33 ± 2.08	5.67 ± 1.53	12.67 ± 2.52	12.33 ± 1.53
	Sotalol	Pharmaceutical	<LOQ	5.33 ± 2.08	3.67 ± 1.53	10.67 ± 2.52	10.33 ± 1.53
	Sulfamethoxazole	Pharmaceutical	<LOQ	4.67 ± 1.53	4.33 ± 2.52	10.67 ± 1.53	10.33 ± 2.52
	Sulfapyridine	Pharmaceutical	<LOQ	3.67 ± 2.08	3.33 ± 2.08	10 ± 1	9 ± 2.65
	Trimethoprim	Pharmaceutical	<LOQ	5 ± 1	4 ± 2.65	11 ± 1	10 ± 2.65
	Venlafaxine	Pharmaceutical	1.33 ± 0.58	5.67 ± 1.53	5.33 ± 2.52	12 ± 1	11 ± 2.65
	Caffeine	Tracer	<LOQ	<LOQ	<LOQ	<LOQ	1.33 ± 0.58
Metals							
	Ag		0.02 ± 0.01	0.03 ± 0.01	0.02 ± 0.01	0.07 ± 0.04	0.02 ± 0.01
	Al		27.21 ± 5.31	52.15 ± 6.76	29.12 ± 1.72	178.86 ± 30.78	40.74 ± 2.83
	Cd		0.01 ± 0	0.02 ± 0	0.01 ± 0	0.04 ± 0.01	0.02 ± 0
	Co		0.21 ± 0.01	0.24 ± 0.01	0.23 ± 0.01	0.37 ± 0.02	0.27 ± 0.03
	Cr		0.6 ± 0.05	0.83 ± 0.06	0.68 ± 0.07	1.7 ± 0.42	0.77 ± 0.08
	Cu		1.83 ± 1.24	3.34 ± 0.52	1.87 ± 0.88	11.9 ± 2.57	1.33 ± 0.35
	Fe		52.59 ± 12.92	49.47 ± 7.29	40.85 ± 7.57	87.24 ± 16.86	28.7 ± 3.25
	Mn		6.96 ± 1.09	5.17 ± 1.7	4.74 ± 0.69	9.96 ± 0.53	2.38 ± 0.77
	Ni		0.85 ± 0.09	0.9 ± 0.06	0.84 ± 0.07	1.47 ± 0.16	0.94 ± 0.08
	Pb		0.21 ± 0.11	0.27 ± 0.04	0.21 ± 0.04	0.75 ± 0.16	0.27 ± 0.04
	Zn		6.34 ± 0.26	21.19 ± 5.58	17.77 ± 4.06	59.67 ± 13.58	42.49 ± 3.24

*Note*: For all types of measured parameters (i.e. abiotic, water quality, micropollutants (MP), and metals), table shows mean and standard deviation per treatment. Measured number of samples per treatment was 60 for abiotic parameters, six for water quality, three for micropollutants, and six for metals. See ‘Section 2’ for further details. Measurements of micropollutants below the limit of quantification are shown as “<LOQ.”

A total of 51 organic micropollutants, consisting of 25 pharmaceuticals, 21 pesticides, two artificial sweeteners, two corrosion inhibitors, and caffeine (i.e. a tracer of sewage effluent in natural waters), were analyzed in grab water samples (Tables 1, S4). These substances were selected for analyses based on their detection frequency, concentration in municipal wastewater, toxicity, analytical restrictions, and substance classes (Munz et al., 2017; Tlili et al., 2017). For analyses of micropollutants, grab water samples (with a 1.4 mL volume) were taken weekly in each channel and stored into 1.5‐mL LC vials at −20°C until analysis. As preliminary results showed negligible variability among replicates (data not shown), micropollutants were finally analyzed only in one channel per treatment. Micropollutant analysis in all samples was performed by HPLC‐MS/MS (see Annex S1 for a detailed description of the procedure).

A total of 11 metals (i.e. Al, Cr, Mn, Fe, Co, Ni, Cu, Zn, Ag, Cd, and Pb) were analyzed in grab water samples (Table S5). The samples (with a 10 mL volume) were taken every week in two replicate channels per treatment, acidified with 100 μL of 65% nitric acid, and stored at 4°C until analysis. Metal quantification was performed by inductively coupled plasma mass spectrometry (8900 Triple Quadrupole ICP‐MS; Agilent).

#### 2.3.2 Host response variables

To estimate the effects of the experimental treatments on host performance at the end of the experiment (after 14 days in the Maiandros), we quantified survival, growth, and food consumption (or feeding). Survival was estimated by counting the number of alive individuals within each experimental container at the end of the experiment. For each class (i.e. males, females, and juveniles), survival was then calculated as the proportion of individuals that were alive out of the total 10 individuals that had been placed per experimental container at the start of the experiment.

Food consumption was quantified as the change in the mean leaf disc area (mean of the five leaf discs per experimental container, in mm^2^) between the start and the end of the experiment. Leaf area (in mm^2^) was calculated from the digital images and measured in FiJi (Fiji by ImageJ, version 2.1.0/1.53c; Schindelin et al., 2012). Growth was quantified as the change in the mean body size between the start and the end of the experiment (mean of the 10 individuals at the start and the number of alive individuals at the end, per experimental container, in mm^2^). Host body size was calculated from the digital images and measured in a semi‐automated manner by using a personalized interactive workflow from *phenopype*; a high‐throughput phenotyping pipeline for *python* (version 1.0.8) (Lürig, 2022). In summary, this workflow consisted of manually drawing a polygon around each individual, which then automatically recognized the body outline (without appendages) by means of thresholding algorithms and segmentation. Body size was extracted as the area in pixels which was later transformed to mm^2^ using the mm to pixel ratio.

#### 2.3.3 Microbiota response variables

For measurements of the gut microbiome in the mid‐ and the hindguts, a subset of two adult males and two adult females per channel were put aside by the end of the experiment. Individuals were placed in six‐well plates and left without food for 15–20 h to allow for clearance of their digestive system. Prior to dissection, animals were surface‐sterilized by performing a wash in bleach (for 8–10 s), followed by two consecutive washes (of 8–10 s each) in Milli‐Q water to remove bleach. Animals were then placed in a sterilized petri dish, with ventral side up, and dissected under a binocular. The dissection consisted of grabbing the anterior‐most and posterior‐most parts of the body with two sterilized forceps and consequently pulling out the guts from the body. The two digestive tissues (i.e. midgut and hindgut) were then stored separately in 1.5 mL Eppendorf tubes, immediately frozen in liquid nitrogen, and preserved at −80°C for later DNA extraction and 16S rRNA amplicon sequencing.

### 2.4 Gut microbiota MiSeq sequencing data

#### 2.4.1 Library preparation

DNA was extracted from the midgut and hindgut tissues using Qiagen DNeasy Blood & Tissue Kit (QIAGEN N.V, Hilden, Germany) according to the manufacturer's instructions. Extracted DNA was stored at −20°C freezer for later processing. A total of 11 gut samples were damaged either during dissection or during subsequent manipulation. Hence, the final number of tissue samples used for 16S rRNA gene amplicon sequencing was 148 samples, corresponding to 71 female samples (35 hindgut and 36 midguts) and 77 male samples (39 hindguts and 38 midgut). Eight reagent‐only DNA extraction negative controls (containing no DNA) and two PCR negative controls (i.e. without template for the indexing‐PCR procedure) were also included. DNA quality was confirmed by running samples in an agarose gel and DNA quantity was measured using Qubit 2.0 (InvitrogenTM). A 445‐bp‐fragment spanning the variable region V3‐V4 of the bacterial 16S rRNA gene was amplified using the universal bacterial primers b341F (5′‐CCTACGGGAGGCAGCAG‐3′, probe S‐D‐Bact‐0341‐a‐S‐17; Klindworth et al., 2013; Muyzer et al., 1993) and 785R (5′‐CTACCAGGGTATCTAATCC‐3′, probe Eco790; Lee et al., 1993). Both primers were adapted for Illumina MiSeq amplicon sequencing library preparation by adding the Nextera adapter, 0–3 bp random frameshifts, and a 19‐bp Multiplex Identifier sequence.

For each sample, three 25‐microliter PCR reactions were performed and pooled. Each 25‐microliter PCR reaction contained 1X Qiagen Multiplex PCR MasterMix, 0.3 μM of both forward and reverse primer, and 3 microliters of template DNA. PCR cycles were performed as follows: 95°C for 15 min, followed by 31 cycles of 95°C for 45 s, 55°C for 60 s, 72°C for 60 s, and a final extension of 72°C for 10 min. PCR products were then subjected to a two‐round purification with AMPure XP beads from Beckman Coulter™. Indexed PCR products were amplified for 10 cycles with Nextera XT v2 indexing primers using KAPA HiFi HotStart ReadyMix (Roche Holding AG, Basel, Switzerland), followed by another two‐step purification with AMPure XP beads. Concentration of each sample library was determined by Qubit (dsDNA‐Assay, Spark 10 M device) prior to normalization. In total, 158 separate libraries were prepared including the (148) tissue samples, eight reagent‐only negative controls from the DNA extraction step, and two reagent‐only controls from the PCR step. The final library pool was quantified using Qubit (Invitrogen) and TapeStation (Agilent Technologies). Pooled libraries were sequenced on an Illumina MiSeq instrument (Illumina) using a 600 cycle v3 sequencing kit and paired‐end 300 sequencing mode at the Genetic Diversity Centre (GDC) Zürich (http://www.gdc.ethz.ch).

We used an amplicon sequence data preparation workflow established by the Genetic Diversity Centre (GDC) on the HPC Euler at ETH Zurich. A detailed log file describing each preparation step can be found in Annex S2. In short, the fastq raw sequence reads were first quality controlled (step A) using Usearch (v11.0.667) to establish the parameters for the workflow. The reads were cleaned (e.g. PhiX removal and low complexity filter), end trimmed (to improve merging) and read pairs were merged (Usearch v11.0.667, step B). In a next step (C) the full‐length primer sites were trimmed from the merged reads (Usearch v11.0.667) followed by a filtering step (e.g. mean quality, GC range, size range) using PRINSEQ‐lite (0.20.4). The filtered amplicon sequences were error‐corrected and clustered with a zero % identity radius (zOTU – Usearch::UNOISE3), and a minimum abundance size of 7. Zero‐radius OTUs (zOTUs) is identical to amplicon sequence variants (ASV) in other workflows. Usearch::SINTAX in combination with SILVA SSU (v128) was used to predict taxonomic associations.

#### 2.4.2 16S rRNA data preprocessing

With an abundance threshold of seven, a total of 11′357 zOTUs were identified. The majority of zOTUs (61%) were rare and had fewer than 100 counts. After additional clustering at 97% similarity, the number of zOTUs (zOTUs_c97) was reduced to 4431 (39%). We used *decontam* R package (Davis et al., 2018) to identify potential contaminants in the negative controls. We used both a frequency and a prevalence approach and did not detect any contaminants. Based on these results, the negative control samples were excluded from the downstream analyses, and the samples were not corrected.

We used the *phyloseq* R package (McMurdie & Holmes, 2013) to perform taxonomic filtering and remove five archaea zOTUs (level Kingdom), 61 Cyanobacteria, 281 Chloroplast, and 44 zOTUs with ambiguous phylum annotation. Prevalence filtering was performed to remove three phyla that appeared only in one sample (i.e. four zOTUs from Fibrobacteres, two zOTUs from Synergistetes, and one zOTU from Thermotogae). Further analyses were done with the remaining 4033 zOTUs. We did not rarefy our data because of the limitations in the analyses (detailed by McMurdie & Holmes, 2014). Moreover, the difference between minimum (32,245) and maximum (164,945) depth was not large (i.e. 5.12×), and there were no imbalances in total counts or number of samples among critical groups (e.g. sexes, tissues, or treatments). However, we did control for differences in sequencing depths in our statistical models of indices of diversity and/or normalize data, as specified below for some analyses.

### 2.5 Statistical analyses

All statistical analyses were conducted in R v 3.6.2 (Team, 2019) using the following R packages: *tidyr* (Wickham & Henry, 2019) to arrange datasets, *ggplot2* (Wickham & Others, 2009) to produce plots, *lme4* (Bates et al., 2014) and *lmerTest* (Kuznetsova et al., 2017) to perform linear mixed‐effects models, *factoextra* (Kassambara & Mundt, 2017) to perform principal components analyses of nutrients, and *phyloseq* (McMurdie & Holmes, 2013), *microbiome* (Lahti & Shetty, 2018), *DESeq2* (Love et al., 2014), *indicspecies* (De Cáceres & Legendre, 2009), and *vegan* (Oksanen et al., 2015) for analyses of microbial diversity and community composition.

#### 2.5.1 Background variables

Abiotic parameters (i.e. pH, temperature, conductivity, and oxygen) were analyzed using generalized linear mixed models to test for the fixed effects of wastewater (WW) (i.e. continuous variable: 0, 30, and 80) and for the interaction between wastewater and ultrafiltration (WW*UF) (i.e. for 30% and 80% WW treatments, ultrafiltration is a fixed factor with two levels: UF and non‐UF). To account for non‐independence of measurements of different individuals within a given channel, channel number was included as a random effect in these models. For all the mixed models, we used Satterthwaite's method for approximating degrees of freedom and estimating *F*‐statistics and *p* values.

Water quality parameters (i.e. nutrients) (Tables 1, S3), micropollutants (Tables 1, S4), and metals (Tables 1, S5) were analyzed using principal components analyses (PCA). For the PCA of micropollutants, we did not consider those compounds that had undetectable levels (i.e. below limit of quantification: < LOQ) in all treatments (Tables 1, S4) (i.e. 2,6‐Dichlorbenzamide, Carbendazim (Azole), Chlortoluron, Diazinon, Dimethenamid, Dimethoate, Diuron, Epoxiconazole, Ethofumesate, Fipronil, Isoproturon, Mecoprop, Metamitron, Metamitron‐Desamino‐4, Metolachlor‐OXA, Pirimicarb, Propiconazole, Tebuconazole, and Terbutryn) (Tables 1, S4). For the remaining micropollutants, we considered values < LOQ equal to zero (when applicable), and we applied a log transformation and a scaling prior to performing the PCA. For all the PCA, we used linear mixed models similar to the ones described above, but using the PC1 as the response variable (Table S6).

#### 2.5.2 Host response variables

Host phenotypes were analyzed using a generalized linear mixed model with the same model structure as described for the background variables (see above), but with the following modifications: (i) size at the start (i.e. mean per experimental unit) was included in all models as a covariate to account for variation in initial body sizes, and (ii) the response variable for the analysis of survival was the proportion of alive/total individuals per experimental unit.

#### 2.5.3 Microbiota response variables

Variation in microbial community composition was estimated using community diversity clustering metrics (i.e. beta‐diversity metrics). To estimate beta‐diversity, we implemented principal coordinate analysis (PCoA) using Bray–Curtis (BC; i.e. dissimilarity based on relative abundance), UniFrac (UNI; i.e. dissimilarity based on presence/absence and phylogenetic diversity), and weighted UniFrac (wUNI; i.e. dissimilarity based on relative abundance and phylogenetic diversity) dissimilarity measures (Legendre et al., 2005; Legendre & Gallagher, 2001). Changes in community composition between sexes (i.e. fixed factor with two levels: females and males), tissues (i.e. fixed factor with two levels: hindgut and midgut), and for the interaction between wastewater and ultrafiltration (WW*UF), as well as interactions among all other fixed factors, were tested on the Bray–Curtis, UniFrac, and weighted UniFrac distances using permutational multivariate analysis of variances (PERMANOVA; adonis), with permutation testing with 999 randomizations. The sequencing depth of each sample and the size of the animals was included as a covariate in the PERMANOVA models.

Global diversity indicators were estimated using Chao1 index (richness estimate), Pielou evenness, Gini index (inequality), and rarity (for low abundance taxa). We used these four diversity estimates as variables in linear mixed effect models with the same model structure as in the aforementioned PERMANOVA. Given the complexity of these models (and the number of interactions), a minimum adequate model was obtained by backward elimination using the Akaike Information Criterion (AIC). Statistical models used for the analyses of beta‐diversity metrics and global diversity indicators per tissue were the same as described above, but excluding the fixed factor “Tissue”. The most prevalent microbial taxa (Neu et al., 2021) were extracted from the samples of the control treatment only (i.e. 0% WW) (via the *core* function of the *microbiome* R package) with a detection threshold of 0.0001 and a prevalence threshold of 0.75.

To assess which taxa contributed the most to the differences among groups, we performed Similarity Percentage analysis (SIMPER) on Bray–Curtis distance matrix (in cases when PERMANOVA was significant; *p* < 0.05) (Oksanen et al., 2015). In order to assess the relative abundance of zOTUs across groups (i.e. tissues, wastewater concentrations, or ultrafiltration treatments), we then applied Kruskal–Wallis tests, with false discovery rate correction <0.05, in all SIMPER zOTUs, except those that individually contributed less than 1% to SIMPER. We also explored differentially abundant zOTUs using *DESeq2*. Indicator species analysis was also performed to identify abundant zOTUs that were specifically associated with wastewater or ultrafiltration treatments by using the *multipatt* function from *indicspecies* R package (De Caceres et al., 2014) with seed set to “270686” and 999 permutations. For the analyses of specific zOTUs differing between ultrafiltration treatments (UF *versus* non‐UF) (i.e. DESeq, SIMPER, and indicator species analyses), we excluded the 0% WW treatment, as no ultrafiltration existed for this concentration of wastewater.

## 3 RESULTS

### 3.1 Effects of the experimental treatments on biotic and abiotic parameters

We had two wastewater (WW) concentrations (i.e. 30% WW and 80% WW) with or without ultrafiltration (UF), and an untreated (and nonfiltered) river water control (0% WW) (Figure 1), which differed in several biotic and abiotic characteristics (Tables 1, S1–S5). Ultrafiltration led to an average (of 33 measures) of 96.5% reduction in bacterial concentrations (Table S1), indicating that the filtration treatment was effective. Treatments also differed in abiotic parameters, reflected in a significant interaction between the wastewater and ultrafiltration for all parameters (Table S2, Figure S1). Overall, pH (WW*UF; *F*_1,17_ = 110.3, *p* < 0.001) and oxygen were higher (WW*UF; *F*_1,17_ = 432.3, *p* < 0.001) in the filtered 80% WW treatment (80% WW‐UF), while temperature (*F*_1,297_ = 144.0, *p* < 0.001) and conductivity (*F*_1,297_ = 17.8, *p* < 0.001) were lower in the filtered 80% WW treatment, than in the nonfiltered 80% WW (Tables 1, S2, S6; Figure S1A–D).

Principal component (PC) analysis of the 16 water quality variables (mostly nutrients) (Tables 1, S3, S6; Figure S1E–G) measured in the treatment waters revealed an effect (on PC1) of wastewater (WW; *F*_1,27_ = 353.6, *p* < 0.001) which was dependent on the presence or absence of ultrafiltration (WW*UF; *F*_1,27_ = 26.5, *p* < 0.001), with overall higher nutrient loading with wastewater and no ultrafiltration (Table 1; Figure S1G). A similar analysis for 51 different micropollutants (Tables 1, S4, S6; Figure S1H–J) showed an effect of wastewater on PC1 (WW; *F*_1,12_ = 95.9, *p* < 0.001), with overall higher concentrations of micropollutants with increasing concentration of wastewater (Table 1; Figure S1J). Finally, a similar analysis for 11 different metals (Tables 1, S5, S6; Figure S1K–M) revealed an effect of wastewater (WW; *F*_1,7_ = 111.8, *p* < 0.001), which was dependent on the presence or absence of ultrafiltration (WW*UF; *F*_1,7_ = 72.9, *p* < 0.001), with an overall increase in metals with higher wastewater concentrations, and especially high concentrations of metals in the 80% WW treatment (Table 1; Figure S1M). Jointly, these results reflect the composite effects of wastewater effluent and indicate that the addition of wastewater altered the physicochemical environment for the experimental organisms.

### 3.2 Host performance is influenced by wastewater

**FIGURE 2:**
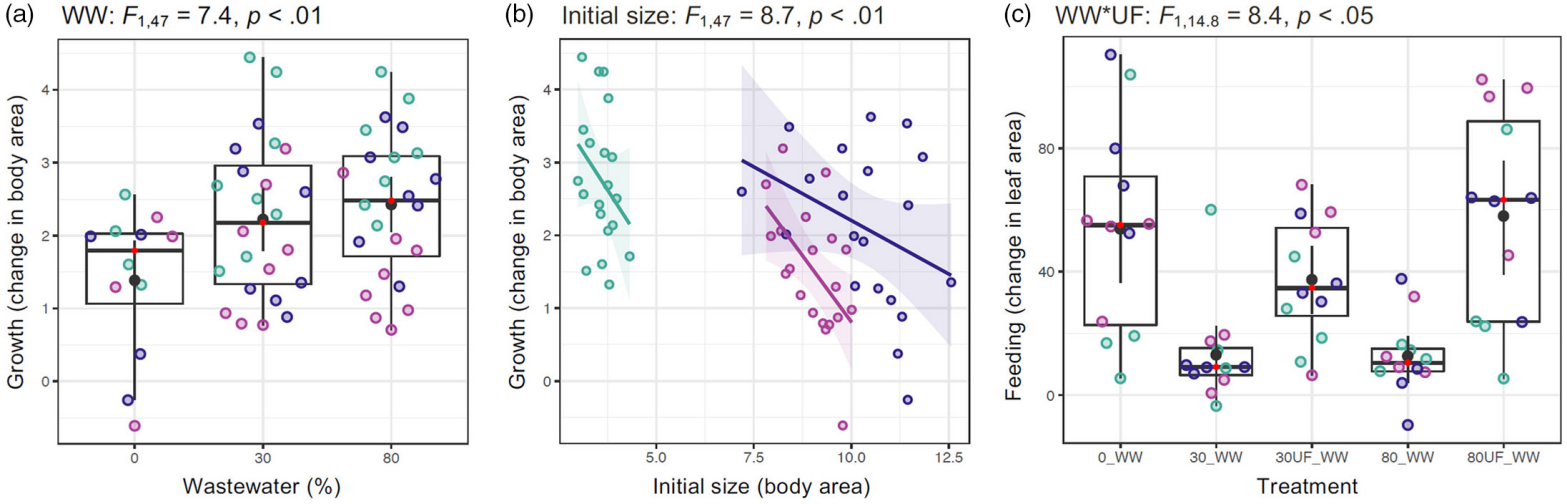
Treatment effects on host performance. (a–c) Influence of wastewater and filtration on host growth and feeding. Juveniles, males, and females are shown with green, blue, and pink points, respectively. (a) Boxplots represent variation for growth, measured as change in mean size (in mm^2^) among wastewater treatments (0%, 30%, and 80% wastewater). (b) Scatter plot representing the relationship between growth and initial size (as mean area, in mm^2^) of *A. aquaticus*. (c) Boxplots represent variation in food consumption (change in mean leaf area, in mm^2^) between nonfiltered wastewater and ultrafiltration (UF: ultrafiltered) treatments (0% WW, 30% WW, 80% WW, 30% WW UF, and 80% WW UF). Statistically significant effects are shown above the plots (for detailed results see Table S6).

In order to assess the combined effects of treated wastewater on host performance and host microbiome, we exposed males, females, and juveniles of *A. aquaticus* to the different treatments for 14 days, after which we quantified host survival and performance (Figure 2; Table S7).

Analyses of host survival showed that neither wastewater concentration (WW; *F*_1,15.9_ = 0.7, *p* > 0.05) nor ultrafiltration (WW*UF; *F*_1,16_ = 0.2, *p* > 0.05) affected host survival. Growth, however, was influenced by the wastewater treatments, being higher, on average, in the presence of wastewater (WW; *F*_1,47_ = 7.3, *p* < 0.01) (Figure 2a). Growth also differed between animals of different starting sizes (Size; *F*_1,47_ = 8.7, *p* < 0.05), with animals of smaller sizes growing relatively more (or faster) independently of the class (Figure 2b). In contrast, feeding was lower with wastewater, but only in the absence of ultrafiltration (WW*UF; *F*_1,14.8_ = 8.3, *p* < 0.05) (Figure 2c).

### 3.3 Gut microbiome composition of mid‐ and hindguts

Bacterial communities were characterized by 16S rRNA sequencing in the midguts and hindguts of a subset of individuals (see ‘Section 2’ and Figure 1). The overall gut bacterial community (both tissues) comprised 4431 zOTUs that corresponded to 22 different bacterial phyla, 82 classes, 123 orders, and 307 families. The most abundant phyla were Proteobacteria (*n* = 1758), Bacteroidetes (*n* = 966), Actinobacteria (*n* = 515), and Firmicutes (*n* = 356), which together accounted for 92% of total abundance. The most prevalent microbial taxa (i.e. zOTUs with prevalence ≥0.75) showed a certain degree of similarity between both tissues (Table S8), with only 10 (out of 71) and 16 (out of 135) taxa being uniquely associated with the midgut and hindgut, respectively (Table S8).

**FIGURE 3:**
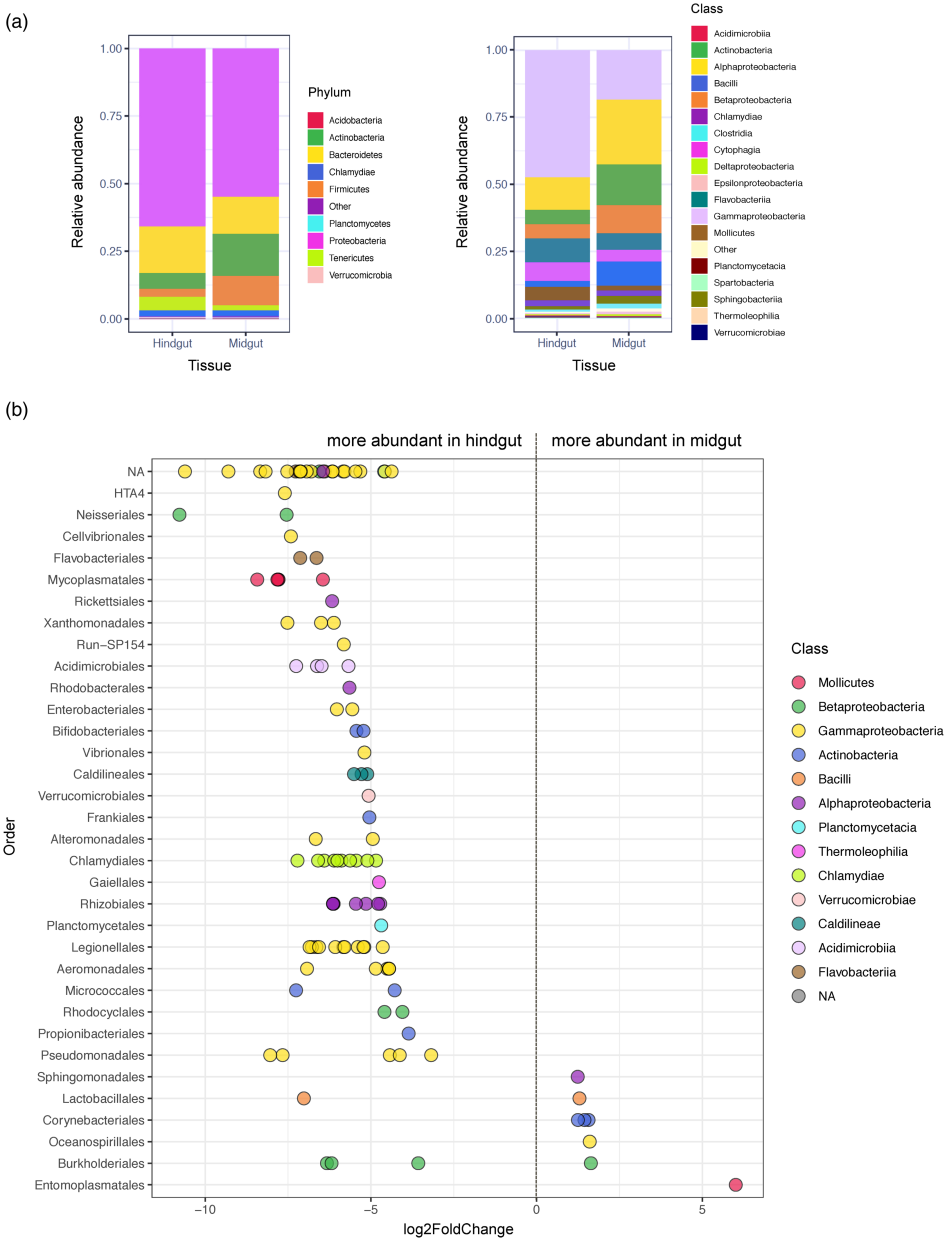
Microbiome composition and abundance. (a) Stacked bar plots show relative abundances of different bacterial taxa (averaged per gut tissue and pooled across treatments) at the level of phylum (left) and class (right). Relative abundances per individual samples can be found in Figure S2I. (b) Differential abundance of operational taxonomic units (zOTUs) between tissues. Dot plot shows those zOTUs that were significantly differentially abundant between gut tissues at the taxonomic level of class (DESeq2; *p* adj <0.001). Dots are colored by the taxonomic order to which each zOTUs belongs to.

Microbial alpha diversity differed between tissues (Table S6, Figure S2A–D), with richness (Chao1; *F*_1,146_ = 16.6, *p* < 0.001) (Figure S2A) and inequality (Gini index; *F*_1,44_ = 7.9, *p* < 0.05) (Figure S2B) being lower in the midgut than in the hindgut. Conversely, evenness (Pielou; *F*_1,146_ = 23.6, *p* < 0.01) (Figure S2C) and rarity (*F*_1,142_ = 6.45, *p* < 0.05) (Figure S2D) were higher in the midgut than in the hindgut (Table S6). Additionally, tissues differed in community composition (Table S6; Figures 3a, S2E–I), as indicated by the significant PERMANOVAs for several dissimilarity metrics (Tissue; adonis_BC_, *F*_1,147_ = 17.2, *p* < 0.001; adonis_wUNI_, *F*_1,147_ = 24.1, *p* < 0.001; adonis_UNI_, *F*_1,147_ = 7.1, *p* < 0.001) (Table S6), and by the clustering per tissue upon ordination analyses (Figure S2E–G). Similarity percentage (SIMPER) analyses on Bray–Curtis distances (Table S9) revealed 14 zOTUs which contributed significantly (after FDR correction) to differences between tissues. For instance, family *Anaplasmataceae* was more abundant in the midgut, whereas families *Aeromonadaceae*, *Vibrionaceae*, and *Shewanellaceae* were more abundant in the hindgut (Table S9). Finally, specific zOTUs were found (via DESeq2 analysis) to differ between tissues (Table S10; Figure 3b), with several zOTUs from orders Mycoplasmatales, Chlamydiales, Rhizobiales, Legionellales, and Pseudomonadales (Figure 3b) having higher abundances in the hindgut than the midgut (Table S10; Figure 3b).

### 3.4 *Wolbachia* impacts bacterial community composition and diversity

The aforementioned ordination analyses of Bray–Curtis dissimilarity metric revealed a cluster of samples (*N* = 30; Figure S2H) with high abundance of zOTU1. This zOTU was classified as *Anaplasmataceae* and likely corresponds to *Wolbachia* (assessed via NCBI Blast; Zhang et al., 2000) *–* a common reproductive manipulator in arthropods (Cordaux et al., 2012; Werren et al., 2008; Weinert et al., 2015), also present in some isopods (e.g. Cordaux et al., 2012).

The second principal component in Bray–Curtis distances (Figure S2H) discriminated between individuals with high abundance of *Wolbachia* (*Wolbachia* abundance; adonis_BC_, *F*_1,147_ = 6.8, *p* < 0.001) in either hindgut and/or midgut – though abundance was higher in the midgut (Table S9) – indicating that the relative abundance of *Wolbachia* influences bacterial community composition in host guts. Taxa contributing the most to differences in community composition between *Wolbachia*‐infected and ‐uninfected individuals (based on SIMPER analyses) belong to genus *Aeromonas*, *Shewanella*, and *Corynebacterium*, all of which were less abundant in *Wolbachia*‐infected than uninfected individuals (Table S9). Bacterial diversity (richness) was also affected by the abundance of *Wolbachia* (Chao1; *F*_1,145_ = 10.9, *p* < 0.01), with lower richness being associated with higher *Wolbachia* abundance (Table S6).

Given the aforementioned effects of *Wolbachia* on bacterial community composition and diversity, reads identified as *Wolbachia* (i.e. zOTU1) were excluded and the relative abundance of *Wolbachia* was included as a predictor in all downstream analyses.

### 3.5 Gut bacterial composition responds to wastewater

**FIGURE 4:**
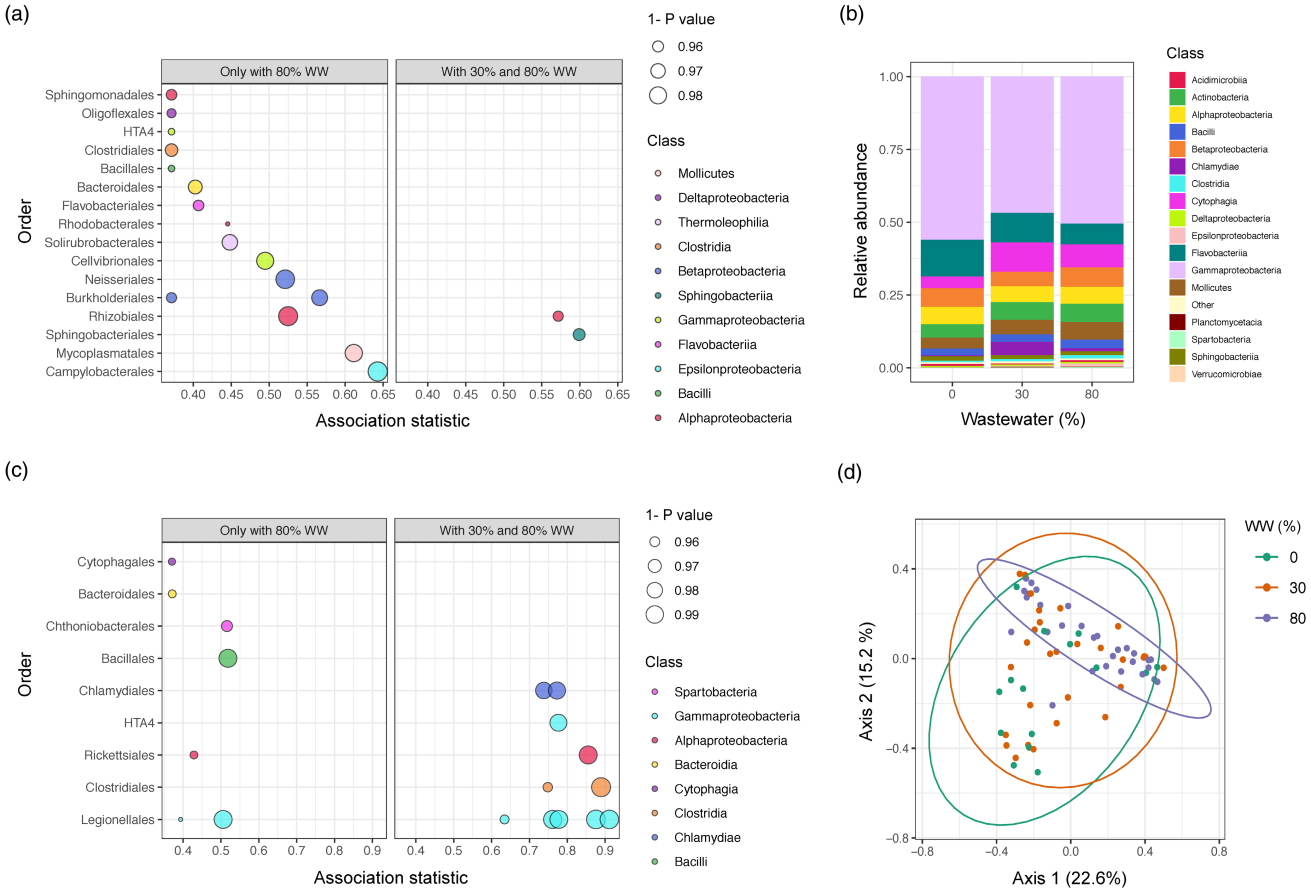
Effects of wastewater on microbiome composition. (a) Indicator taxa in different wastewater concentrations in the hindgut. Dot plot shows significant zOTUs (*p* adj <0.05) at the taxonomic level of class. Dot size represents the *p* value, and dot color represents the taxonomic order to which each zOTUs belongs to. (b) Stacked barplots show relative abundance averaged per wastewater concentration (0%, 30%, or 80%WW) of different bacterial taxa at the level of class in the hindgut. Relative abundances per individual samples can be found in Figure S3A. (c) Indicator taxa in different wastewater concentrations in the midgut. Dot plot shows every significant zOTU (*p* adj < 0.05), at the taxonomic level of class. Dot size represents the *p* value, and dot color represents the taxonomic order to which each zOTU belongs to. (d) Ordination plot of the principal coordinate analysis (PCoA) for hindgut samples based on Bray–Curtis (BC) distances colored and grouped by treatment, with 0%, 30%, and 80% wastewater in green, orange, and purple, respectively. Ellipses denote the 95% confidence interval.

Analyses of bacterial diversity indicated that wastewater did not influence richness, evenness, inequality, nor rarity in neither the mid‐ nor the hindgut (Table S6). However, wastewater contributed to variation in microbial community composition in the hindgut (Tables S6–S11; Figure 4), as described below.

*Midgut* – Wastewater did not affect bacterial diversity nor community composition in the midgut (WW; *p >* 0.05 for all diversity and dissimilarity metrics) (Table S6). Only two zOTUs appeared to differ in abundance (via DESeq2) between the wastewater treatments and corresponded to taxa from families *Staphylococcaceae* and *Fusobacteriaceae* (Table S10). Potential indicator taxa, responding positively to both 30% and 80% wastewater input in the midgut, belonged to families *Phyllobacteriaceae* and *NS11‐12 marine group* (Figure 4a; Table S11). Several other (17) zOTUs responded only to high wastewater concentration, including taxa from families *Arcobacter*, *Candidatus Bacilloplasma*, and *Cryomorphaceae* (Figure 4a; Table S11).

*Hindgut* – Wastewater affected overall bacterial community composition in terms of relative abundances of taxa in the hindgut (WW; adonis_BC_, *F*_1,73_ = 5.1, *p* < 0.001) (Figure 4b, Figure S3A). However, this effect was only marginal when accounting for phylogenetic relatedness between taxa (WW; adonis_wUNI_, *F*_1,73_ = 2.2, *p* = 0.04; adonis_UNI_, *F*_1,73_ = 1.3, *p* > 0.05) (Table S6). Ordination analysis of Bray–Curtis distances further revealed a clear cluster reflecting differences between the 80% and the 0% and 30% wastewater treatments (Figure 4d). Decomposition of Bray–Curtis distances (via SIMPER) revealed three zOTUs that decreased in abundance in the presence of wastewater (after FDR correction, Table S9). These three zOTUs included one taxa within the family *Vibrionaceae* and two other taxa within the genus *Pseudomonas* and *Flavobacterium*, respectively (Table S9).

At the level of changes in specific zOTUs between treatments, we found eight zOTUs differentially abundant (via DESeq2) between wastewater treatments (Table S10). Of these, zOTUs belonging to the genus *Candidatus Bacilloplasma*, *Deefgea*, and *Legionellaceae* increased in abundance with both 30% and 80% wastewater, while two zOTUs from *Mycoplasmataceae* increased in abundance only with 80% wastewater. Furthermore, one zOTU from the family *Vibrionaceae*, as well as two nonidentified zOTUs belonging to *Gammaproteobacteria*, decreased with wastewater (Table S10). Finally, potential bacterial indicator taxa that increase with wastewater input in the hindgut (Table S11) included several zOTUs from families *Legionellaceae*, *Clostridiaceae*, and *Holosporaceae*, and from order Chlamydiales, which responded to both lower (i.e. 30% WW) and higher (i.e. 80% WW) concentrations of wastewater (Table S11; Figure 4c). In addition, several taxa from families *Staphylococcaceae* and *Bacteroidaceae*, and order Cytophagaceae, responded only to high concentration of wastewater (i.e. 80% WW) (Table S11; Figure 4c).

In summary, wastewater did not influence gut bacterial diversity, but had an effect on overall community composition of the hindgut, with bacteria from different taxa decreasing in abundance in the presence of wastewater. At the level of specific zOTUs, different taxa responded to wastewater in both mid‐ and hindguts.

### 3.6 Ultrafiltration influences gut microbiome diversity and composition

**FIGURE 5:**
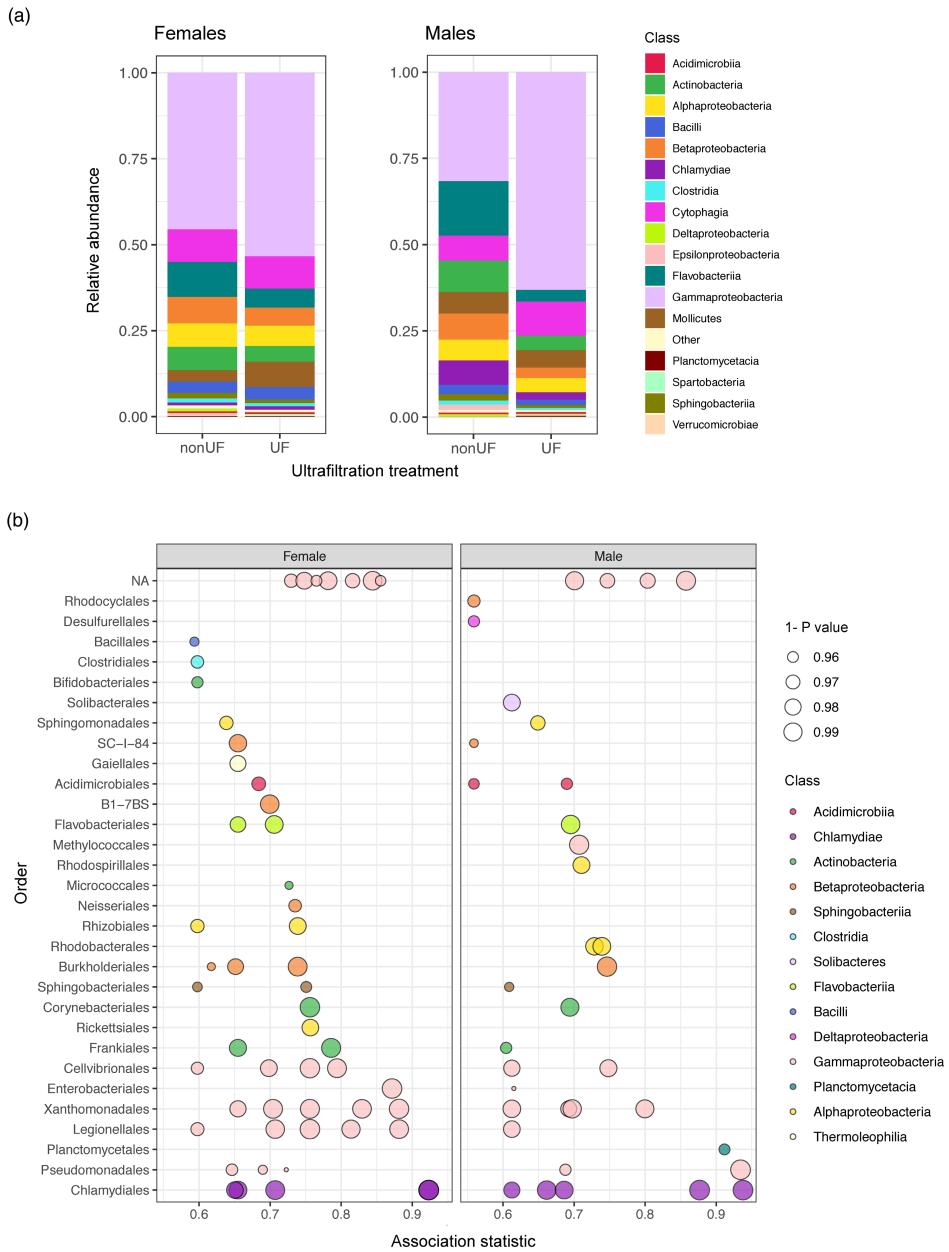
Effects of wastewater‐associated bacteria on microbiome composition. (a). Stacked bar plots show relative abundances of different bacterial taxa (averaged per ultrafiltration (UF) treatment) of different bacterial taxa at the taxonomic level of class in males (left plot) and females (right plot). Wastewater treatments without ultrafiltration (i.e. non‐UF: 30%WW and 80%WW) and with ultrafiltration (i.e. UF: 30%WW‐UF and 80%WW‐UF) are pooled in these plots. The relative abundances per individual samples can be found in Figure S1B. (b) Indicator taxa associated with the ultrafiltration (UF) treatments (pooled across different WW dilution treatments) in the hindguts of females and males. Dot plot shows every significant zOTUs (*p* adj < 0.05), at the taxonomic level of class. Dot size represents the *p* value, and dot color represents the taxonomic order to which each zOTUs belongs to.

We found an effect of ultrafiltration on bacterial diversity as well as on several community composition metrics on the hindgut, but not on the midgut (Table S6). These effects were dependent on sex and wastewater concentration (i.e. significant sex*WW*UF interaction) (Tables S6–S11; Figure 5), as described below.

*Midgut* – In the midgut, ultrafiltration did not affect bacterial diversity nor community composition (WW*UF; *p >* 0.05 for all diversity and dissimilarity metrics) (Table S6). At the level of specific zOTUs, three were differentially abundant (via DESeq2) between ultrafiltration treatments and were identified as *Candidatus Hepatoplasma*, *Candidatus Nitrotoga*, and one more zOTU belonging to order Sphingobacteriales (Table S10). Another 25 zOTUs increased with the ultrafiltration treatment (via indicator species) (Table S11) and included multiple zOTUs from orders Rhizobiales, Propionibacteriales, Flavobacteriales, and Sphingomonadales (Table S11).

*Hindgut* – In the hindgut, ultrafiltration influenced bacterial diversity, an effect that was dependent on sex and on wastewater concentration (sex*WW*UF; Gini index: *F*_1,66_ = 4.8, *p* < 0.05; Rarity: *F*_1,66_ = 5.2, *p* < 0.05). Further exploration of diversity indices per sex revealed that in females, but not in males, evenness differed between treatments (WW*UF; Pielou index: *F*_1,36_ = 5.2, *p* < 0.05), with higher evenness in 80% wastewater (Figure S3C) (Table S6). Moreover, community composition was affected by ultrafiltration in a sex‐specific and wastewater‐concentration manner (sex*WW*UF; adonis_BC_, *F*_1,73_ = 1.8, *p* < 0.05; adonis_wUNI_, *F*_1,73_ = 2.4, *p* < 0.05; adonis_UNI_, *F*_1,63_ = 1.4, *p* < 0.05) (Figures 5a, S3B). Decomposition of Bray–Curtis distances (via SIMPER) revealed that in females, no zOTU differed (after FDR correction) in abundance with ultrafiltration, but one did in males (Table S9). This zOTU corresponded to *Candidatus Bacilloplasma*, which decreased in abundance with ultrafiltration (Table S9).

At the level of changes in specific zOTUs between treatments (via DESeq2), differential abundances between ultrafiltration treatments included seven zOTUs in females (e.g. from families *Flavobacteriales* and *Parachlamydiaceae*) and only two zOTUs (from genus *Pseudomonas*) in males (Table S10). Indicator taxa that responded to ultrafiltration revealed an overrepresentation of taxa from orders Chlamydiales, Pseudomonadales, Legionellales, Xanthomonadales, and Burkholderiales with multiple zOTUs, which differed between the sexes in their relative abundances (Figure 5b, Table S11).

## 4 DISCUSSION

Anthropogenic activities commonly result in a complex suite of biotic and abiotic stressors, but the effects of complex stressors on natural populations, and on host–microbiome interactions in particular, are poorly understood. Here, we used a semi‐natural flume experiment to investigate the effects of nonfiltered (i.e. a mixture of chemical contaminants, microbes, nutrients, and organic particles) *versus* ultrafiltered (i.e. a mixture of dissolved chemicals) wastewater on a keystone detritivore host (*Asellus aquaticus*) and its gut microbiome community. In terms of effects on the host, we found that *A. aquaticus* individuals grew better in the presence of wastewater but fed less (on standardized food provided) in the presence of nonfiltered wastewater, suggesting that wastewater provided the host with additional food sources. In terms of host‐associated microbiomes, we found that both wastewater and dissolved chemicals influenced gut bacterial community composition and the relative abundance of several specific taxa and that these responses showed tissue and sex specificity.

### 4.1 Effects of wastewater and dissolved chemicals on host performance

We found higher growth in the presence of wastewater (regardless of the age class and the ultrafiltration treatment) but reduced feeding rates of *A. aquaticus* in the presence of nonfiltered wastewater (compared to unmanipulated river water). As wastewater effluent is a complex mixture of nutrients, microbes, micropollutants, and organic particles and differs also in temperature and chemical properties (Stamm et al., 2016) (Figure S1), the pathways influencing the performance of *A. aquaticus* are likely to be multifarious. These range from direct effects of temperature (higher in wastewater) on the ectotherm host to the indirect effects via environmental microbes. The latter can happen due to elevated nutrient and toxicant levels in wastewater influencing microbial abundance and community composition via waterborne exposure or via dietary pathways (e.g. affecting food abundance or quality) (e.g. Konschak et al., 2020). Moreover, wastewater effluents serve as a direct input of microbes to the receiving ecosystem, including for instance, human‐associated pathogens such as *Escherichia coli* (e.g. Anastasi et al., 2012).

Our ultrafiltration treatment, which removed ~97% of microbes and wastewater‐related organic particles, allows some insight into putative drivers of host responses. The observation that growth increased in the presence of wastewater regardless of the ultrafiltration treatment suggests that either the dissolved pollutants or nutrients (i.e. the two components common to nonfiltered and ultra‐filtered treatments) might be driving those effects on host growth. In this regard, previous studies have reported higher growth rates for *A. aquaticus* feeding on phosphorus and nitrogen‐rich diets (Rossi & Fano, 1979; Graça et al., 1993; Lürig & Matthews, 2021), both of which were elevated in our study in the presence of wastewater (Tables 1, S3). It is, however, unlikely that the observed increase in growth was due to the presence of more nutritious food in wastewater, as this should have resulted in an even higher growth in nonfiltered wastewater – containing higher levels of several nutrients – that we did not observe. It is therefore likely that the effects of wastewater on growth were mediated by chemical pollution (i.e. micropollutants), though the causal effects require further study. Higher growth under chemical pollution could be a generic stress response, via for instance hormesis or compensatory growth (both of which can lead to increased growth due to disturbance; Hornick et al., 2000; Yearsley et al., 2004) or reflect the effects of specific pollutants, such as certain pharmaceuticals, which can affect host physiology (Heath, 2018; Rhind et al., 2010). Furthermore, chemical pollutants may have influenced the dietary food sources of *A. aquaticus* (Feckler et al., 2016). Interactive effects between different wastewater components are also possible. Previous work on *A. aquaticus* shows that while pollutants (e.g. pesticides) alone may not impact organismal survival, the combination of pesticides with other stressors (e.g. predation) can increase mortality (Bundschuh et al., 2012). Such interactive effects of stressors on host performance traits are likely in our study, given the multifactorial nature of our stressor (i.e. treated wastewater).

Interestingly, and somewhat counterintuitively – despite the positive effect of wastewater (independent of ultrafiltration) on *A. aquaticus* growth – food intake of the provided standardized leaf discs was reduced in the presence of nonfiltered wastewater. It is possible that in the nonfiltered wastewater treatments, *A. aquaticus* was feeding on available particulate organic matter and microbes rather than on the leaf discs provided in the experimental containers. Even though the main food source of *A. aquaticus* is considered to be leaf litter and detritus (e.g. Graca et al., 1993b), the species is known to also feed on nutritional sources provided by a range of microbes, including bacteria, algae, and fungi (Bloor, 2011; Graça et al., 1993), with microbially enriched substrates being even preferred (Bohmann, 2005; Graca et al., 1993a; Marcus et al., 1978). This microbial composition of the diet can have a substantial impact on *A. aquaticus* performance by, for example, providing essential fatty acids (e.g. Grieve & Lau, 2018).

### 4.2 Effects of wastewater and dissolved chemicals on gut microbiome

Besides influencing host performance, our results showed that nonfiltered wastewater affected gut bacterial composition (but not diversity), while ultrafiltered wastewater affected both bacterial diversity and composition of the hindgut in a sex‐dependent manner (covered in the next section). Chemical pollutants in wastewater represent an important threat to aquatic ecosystems (Gessner & Tlili, 2016; Mateo‐Sagasta et al., 2017; Vörösmarty et al., 2010) and can affect freshwater organisms (Luan et al., 2020; Peschke et al., 2014). However, to date, only few studies have tried to disentangle the relative contributions of the different wastewater components on microbial communities (see e.g. Carles et al., 2021; Tamminen et al., 2021). Moreover, previous studies have investigated responses in environmental (e.g. Tamminen et al., 2021), but not host‐associated microbiomes (but e.g. Mehl et al., 2021). Our data provide rare insight into differential effects of wastewater and dissolved chemicals on host‐associated microbiomes. In particular, in the presence of wastewater, several bacteria (with putatively diverse functions; see below) changed in abundance in the mid‐ and hindgut tissues of the host. The effects of wastewater on bacterial composition were weaker in the midgut than in the hindgut (i.e. no effects on overall community composition in the midgut and only changes in abundance of specific zOTUs), potentially indicating that the hindgut may be more influenced by environmentally acquired (e.g. from diet) microbes than the midgut. We return to specific taxa and their putative functional roles in the section below.

Similar to what we described earlier for effects on hosts, the changes in the host‐associated microbial community in response to environmental conditions (i.e. wastewater constituents) can result from different processes. On the one hand, it is possible that the effect of wastewater on gut microbiomes could arise if chemical stress changed host physiology and this, subsequently, affected the gut microbiome (e.g. Stothart et al., 2019). On the other hand, it is also possible that wastewater affected the gut microbiome by influencing the environmental bacteria available for the host to acquire, either as part of their dietary resources or as acquired symbionts (e.g. Yang et al., 2021). For instance, wastewater effluents can influence microbial communities in the receiving water bodies (e.g. by releasing adapted microorganisms), as well as their ecological function (such as their biotransformation potential; e.g. Desiante et al., 2022), and, thereby, potentially influence environmentally acquired microbiomes of the host (e.g. Mehl et al., 2021).

In the case of *A. aquaticus*, the mechanisms of acquisition (and assemblage) of the gut microbiome are still unknown, but it is likely that a proportion of the bacterial community is gained from the environment, as seen in other animals (Mulder et al., 2009; Maki et al., 2020; Nishino et al., 2021), such as corals which can selectively uptake specific beneficial bacteria taxa (Hoadley et al., 2021). Several taxa that we found in large numbers in the guts of *A. aquaticus* (e.g. *Legionella* or *Pseudomonas*) are often present in high abundances in wastewater (Caicedo et al., 2019; Rizzo et al., 2013), potentially indicating that at least part of the host microbiome could reflect the composition (and abundance) of microbes in the water column. Recent studies on bacterial taxa in (Swiss) rivers impacted by wastewater (Tamminen et al., 2021) found no specific bacterial taxa to be associated with wastewater, but some of the groups (e.g. unidentified *Rhodobacter* species) which we found to be associated with wastewater in *A. aquaticus* gut microbiome (Table S11) were negatively associated by micropollutants in their study. The lack of data on environmental microbiomes in our study does not allow us to assess the extent to which the observed patterns (in bacterial abundances) in the *A. aquaticus* gut reflect variation in abundance of environmental microbes and/or changes in host‐associated microbes. Future studies should assess the (potential) functional relevance of changes in specific bacterial taxa and the extent to which they reflect environmental abundance or functional host association.

### 4.3 Sex‐specific microbiome effects

Our study revealed that the presence of ultrafiltered wastewater (i.e. with dissolved chemicals but not microbes nor organic particles) affected the diversity and the composition of the hindgut bacterial community in a sex‐dependent manner. These effects suggest that dissolved chemicals (in particular micropollutants) *versus* microbes and/or organic particles on host microbiome can be sex‐specific. The ultrafiltration treatment simultaneously reduced microbial loading and the abundance of organic matter and thereby seemed to alter *A. aquaticus* food sources (discussed above) and the physicochemical properties of the ultrafiltered water. Hence, while it is likely that many of the effects on *A. aquaticus* gut microbiome are due to experimental removal of a majority of microbes (i.e. in ultrafiltered wastewater), other pathways are possible.

Our results indicate that wastewater effluents can substantially affect ecological function (e.g. leaf litter degradation) of a keystone detritivore. Importantly, the sexual dimorphism in responses to ultrafiltered wastewater found in this system suggests that females and males may have different susceptibilities to the same stressor which, when influencing host performance, could lead to persistent differences between sexes. While sexual dimorphism is a common feature in a broad range of taxa in nature (Hedrick & Temeles, 1989; Shine, 1989; Zarkower, 2001), sexual dimorphism in the microbiome composition (and its response to stressors) has thus far been largely overlooked in natural populations in general, and aquatic taxa in particular (Bates et al., 2022), as many studies do not include different sexes (but e.g. Góngora et al., 2021; Markle et al., 2013; Valeri & Endres, 2021). Targeted studies exploring sexual dimorphism in host‐associated microbiomes can help us understand the drivers and the consequences of sex‐specific changes as well as their potential to influence population dynamics and selection.

The importance of sex‐specific microbial associations, and their putative consequences for population dynamics, is well illustrated by host‐associated bacterial taxa that influence host reproduction. This is the case of *Wolbachia*, a maternally inherited endosymbiont that is a reproductive manipulator in a range of arthropod taxa (Charlat et al., 2003; Cordaux et al., 2012; Hilgenboecker et al., 2008), including isopods (Dittmer & Bouchon, 2018), and that was highly prevalent in *A. aquaticus*. The gut microbiome of *A. aquaticus* infected with *Wolbachia* differed both in diversity and composition from that of uninfected individuals. While we cannot currently assess whether *Wolbachia* indeed caused the observed changes or whether certain microbiome composition (or other correlated host characteristics) led to *Wolbachia* infections being more likely in certain individuals, studies on other taxa, including terrestrial isopods (Dittmer & Bouchon, 2018), have shown that *Wolbachia* infections can affect the abundance and diversity of microbial communities (e.g. grasshoppers; Duan et al., 2020, mosquitoes; Audsley et al., 2018). The role that *Wolbachia* plays in *A. aquaticus* populations and/or sex determination has not been studied to date and provides a future avenue of research. Such studies on *A. aquaticus‐Wolbachia* associations and on sex‐specific microbial effects could provide an exciting aquatic model system to explore eco‐evolutionary processes via hosts and their associated microbiomes.

### 4.4 Potential functional consequences of changes in gut microbiome

The gut microbiome of *A. aquaticus*, and of isopods in general (Bouchon et al., 2016), is known to play crucial digestive functions (Zimmer & Bartholmé, 2003), and, thus, changes in its composition have the potential to influence host performance (unless different taxa are functionally redundant; Estrada‐Peña et al., 2020; Tian et al., 2020). Midgut (or hepatopancreatic) bacterial symbionts in particular are thought to play a key role in the utilization of low‐quality food sources (Zimmer & Bartholmé, 2003). In agreement with previous studies (e.g. Wang et al., 2007), we found that *A. aquaticus* hosts a diverse gut microbiota, with many of the taxa likely contributing to digestion of challenging food sources (e.g. lignocellulose and chitin; Bredon et al., 2020; Zimmer & Bartholmé, 2003) as well as providing nutritional resources, such as fatty acids (Doroszkiewicz et al., 2021). This includes bacterial taxa with potentially dietary functions, such as *Flavobacterium* or *Pseudomonas* (highly prevalent in *A. aquaticus*) and *Rhodobacters* or *Alcaligenes*, that may aid digestion (Dailey et al., 2016; Yao et al., 2018) or produce compounds of nutritional value (e.g. amino acids, fatty acids; Dailey et al., 2016; Yao et al., 2018). The effects that wastewater and dissolved chemicals had on some of these taxa could therefore influence the host digestive function. For instance, Flavobacteria (decreasing in abundance with wastewater; Table S9) includes species known to degrade cellulose derivatives and plant and fungi components (Cortes‐Tolalpa et al., 2018; Reyes & Jm, 1976; Herrera et al., 2019), common dietary sources of *A. aquaticus* (Graça et al., 1993). It is however important to note that the wild‐collected isopods used in this study were maintained under controlled laboratory conditions (for ∼2 weeks) before the treatment exposure (see ‘Section 2’), and, hence, their native microbiome composition may have been altered prior to experimental set up. Future studies, where the gut microbiome of isopods is sampled directly upon collection from their natural habitat, could shed light onto the generality of our findings with regard to which taxa change in response to wastewater treatments.

Besides the dietary function, the gut microbiota might play a detoxifying role (Ceja‐Navarro et al., 2015; Turner & Bucking, 2019) and therefore influence host response to chemicals. We still know little about the role of gut microbes in the context of chemicals in wastewater, but several lines of evidence suggest that host‐associated gut microbes could have a particularly relevant role in detoxification for the hosts. Notably, some bacterial taxa have an extensive capacity to metabolize environmental chemicals (Ceja‐Navarro et al., 2015; Claus et al., 2017; Gao et al., 2010; Monroy‐Torres et al., 2019). For instance, strains from genus *Shewanella* (abundant in *A. aquaticus*) are resistant to toxic pollutants and/or able to detoxify deleterious compounds (Lemaire et al., 2020). Such bacteria‐dependent metabolism of pollutants could modulate the toxicity for the host (e.g. Daisley et al., 2018). Moreover, environmental contaminants can alter the composition and/or the metabolic activity of gut bacteria (e.g. by inhibiting bacterial growth or inducing dysbiosis; Kish et al., 2013; Rosenfeld, 2017) and subsequently affect host‐associated bacteria and their responses to pollutants. Hence, the gut microbiome can be an important, but thus far underestimated, element that should be considered to fully understand the toxicity of environmental pollutants for the host (Claus et al., 2017).

Wastewater exposure in *A. aquaticus* could also have negative consequences – as suggested by the observed increase in abundance of potentially pathogenic bacteria, such as *Legionella*, *Clostridium*, or *Pseudomonas* (changing with nonfiltered wastewater) and Chlamydiales (increasing with ultrafiltered wastewater). It is conceivable that *A. aquaticus* could serve as a vector for pathogenic bacteria, as seen in some other invertebrates (Alonso et al., 1999; Brassinga et al., 2010; Gismervik et al., 2014). In this regard, the role of *A. aquaticus* and of other organisms as potential carriers of bacteria into drinking water systems (where *A. aquaticus* has been reported; Christensen et al., 2013; Gunkel et al., 2021; Levy et al., 1986) can be of concern.

Despite those putative bacterial functional effects, it is not easy to draw conclusions solely on the basis of taxonomic diversity since many, if not most, bacterial genera have great metabolic versatility and have evolved both pathogenic and beneficial interactions with their hosts (Eloe‐Fadrosh & Rasko, 2013; Hurst, 2017). This includes some of the aforementioned bacterial taxa, such as *Pseudomonas*, highly prevalent in *A. aquaticus* (Table S8) and common in isopod digestive systems in general (e.g. Ullrich, 1991). *Pseudomonas* is a genus that show pathogenic and commensal interactions with their hosts (Crone et al., 2020; Saati‐Santamaría et al., 2021; Silby et al., 2011), the latter including degradation of cellulose (e.g. Palleroni, 1981), nitrogen fixing (e.g. van Borm et al., 2002), and biodegradation of plastics (e.g. Kim et al., 2020). Similarly, while *Clostridia* are best known for their pathogenicity in humans, some species can provide the host with probiotic effects (e.g. Guo et al., 2020) or resistance to pollutants (e.g. herbicides; Shehata et al., 2013). These functional differences can happen at the level of strains (Moore et al., 1998), even when having identical 16S rRNA gene sequences (Jaspers & Overmann, 2004), a level of resolution that we could not obtain with our data.

Future studies trying to understand the role of the host‐associated microbiome on coping with stress, and in particular with wastewater, would benefit from including functional assessments of bacterial taxa, comparing the enzymatic and/or metabolic activities of candidate taxa in stress responses (Gray & Head, 2001; Moore et al., 1998), as has been done with other microbial communities changing in response to wastewater (e.g. with river biofilm; Desiante et al., 2022).

### 4.5 Conclusions and general implications

Taken together, our results show that both hosts and host‐associated microbiomes were affected by wastewater, indicating that this widespread and complex anthropogenic stressor has the potential to influence organismal performance and population persistence in nature (e.g. at point‐source discharges from wastewater treatment plants). Such effects of anthropogenic changes in general, and wastewater in particular, can be particularly striking when they affect keystone species, such as the detritivorous *A. aquaticus* (discussed in Lafuente et al., 2021), as they have the potential to cascade through influences on multiple trophic levels (e.g. on their mesopredators and/or parasites), as well as through nutrient cycling and decomposition (Feckler et al., 2016; Salo et al., 2017; Stamm et al., 2016).

Furthermore, we observed differential responses to (nonfiltered) wastewater and dissolved chemicals in the absence of microbial or organic particle input (ultrafiltered wastewater), suggesting that host–microbiome responses to wastewater result from the combined effects of chemical pollution and microbial loading (in addition to input of nutrients and organic particles). Previous studies on aquatic systems, focusing primarily on host but not host–microbiome responses, have shown that single stressors and composite wastewater can impair biodiversity (Burdon et al., 2016, 2020; Reid et al., 2019; Salo et al., 2017) and that interactions between multiple stressors (e.g. high temperature and pollution) can either amplify or mitigate the impact of individual stressors (e.g. when having opposite effects; e.g. Salo et al., 2017). Our study also shows that composite effects of wastewater can substantially differ from those of chemicals and/or microbial loading, which may have strong ecological consequences via host function and their microbiomes.

Understanding the consequences that human activity has on natural populations and ecosystems, particularly detrimental in freshwater habitats (e.g. Harrison et al., 2019; Reid et al., 2019), requires more empirical examples investigating host–microbiome interactions. While numerous studies have investigated how environmental changes affect organismal performance (Lamma, 2020) and/or community composition (Hammond et al., 2020), the joint responses of host and host microbiome are still poorly understood (Koskella et al., 2017). Future studies exploring how microbiome function is influenced by anthropogenic stressors, such as wastewater, will help elucidate the role of microbiomes in host responses to stress and in evolutionary trajectories of natural populations.

## CONFLICT OF INTEREST STATEMENT

The authors declare no competing interest.

## Supporting information


Tables S1.

Figure S1.

Annex S1.

Annex S2.

## Data Availability

The data for this study are available in supplementary material or have been deposited in the European Nucleotide Archive (ENA) at EMBL‐EBI under accession number PRJEB60404.
