# Supplementary material for: Effects of anthropogenic stress on hosts and their microbiomes: Treated wastewater alters performance and gut microbiome of a key detritivore (Asellus aquaticus)
Source: Evol Appl. 2023 Mar 30;16(4):824–48. doi: 10.1111/eva.13540 (PMC10130563; doi:10.1111/eva.13540)
Supplement: Supplementary file 2 — Figure S1. [file EVA-16-824-s004.pdf]

SUPPLEMENTARY FIGURES

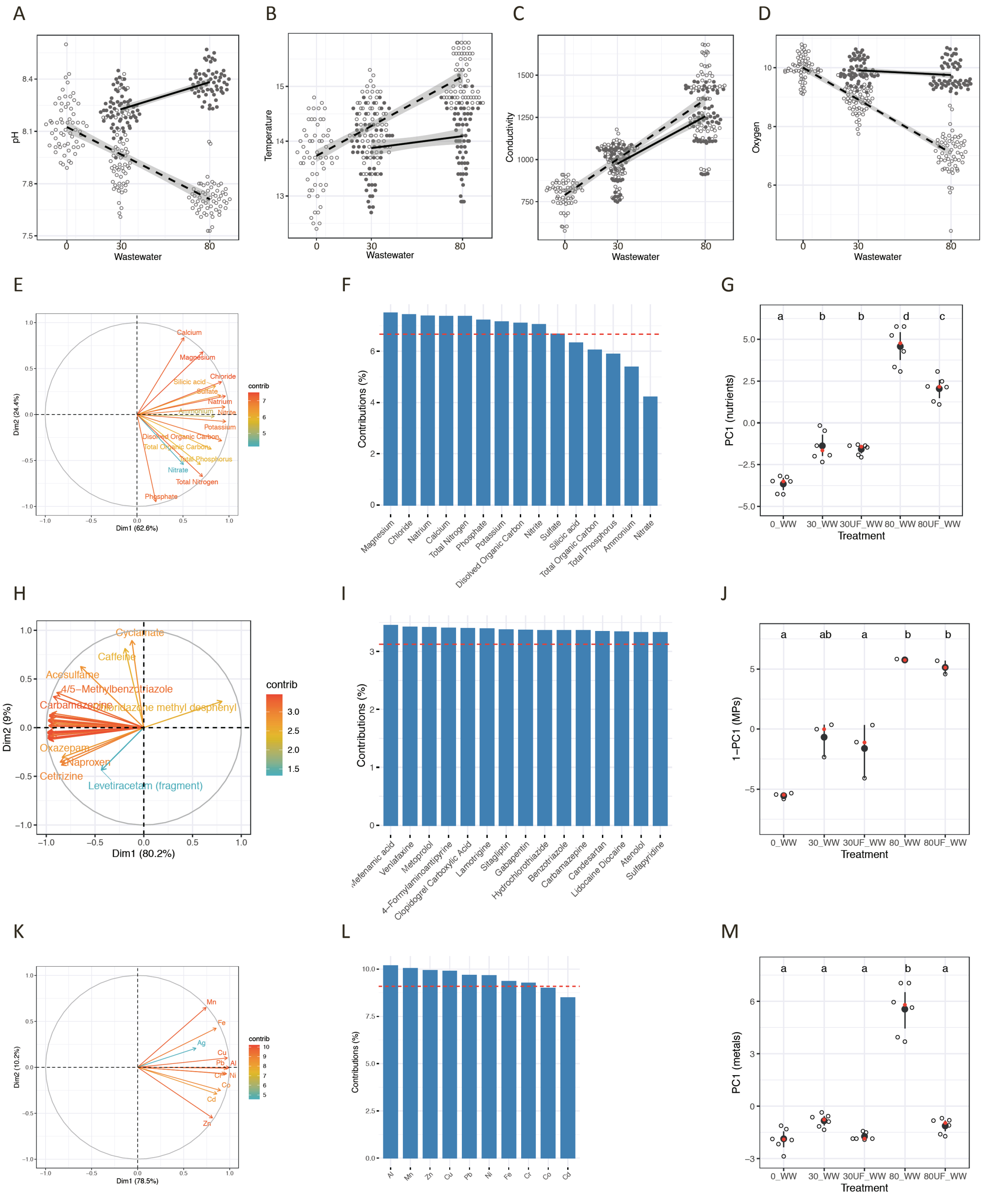

Suppl. Fig. 1. Effects of wastewater and ultrafiltration on abiotic and biotic parameters

A-D: Differences in abiotic parameters between treatments. **E-G:** Differences in nutrients between treatments. **H-J:** Differences in micropollutants between treatments. **K-M:** Differences in metals between treatments. In **A-D**, interaction plots show the predicted values of pH (**A**), oxygen (**B**), conductivity (**C**), and temperature (**D**) in different wastewater treatments (x axis: % WW) and with (solid line, filled circles) or

without (dashed line, empty circles) ultrafiltration. In **E-G, E**: results of PCA with different nutrients indicated by arrows, which indicate their 'weight' in different directions, **F:** Bar plot showing contribution of different nutrients to the first and second components (i.e. dimensions) of the PCA and **G**: violin plot showing the PC1 for nutrients in each of the treatments. In **H-J, H:** results of PCA with different micropollutants indicated by arrows, which indicate their 'weight' in different directions. (Note that nearly-overlapping arrows do not show the name of the micropollutant), **I:** Barplot showing contribution of 15 micropollutants that contributed the most to the first and second components of the PCA. **J**. Violin plot showing the PC1 for micropollutants in each of the treatments. In **K-M, K.** shows results of PCA with different metals indicated by arrows, which indicate their 'weight' in different directions, **L.** Bar plot shows the contribution of different metals to the first and second components of the PCA, **M**. Violin plot shows the PC1 for metals in each of the treatments. In plots **G, J,** and **M,** Tukey's post hoc honest significant differences (for linear model testing effect of *Treatment* on *Parameter PC1*; P < .01) are indicated by different letters in each plot.

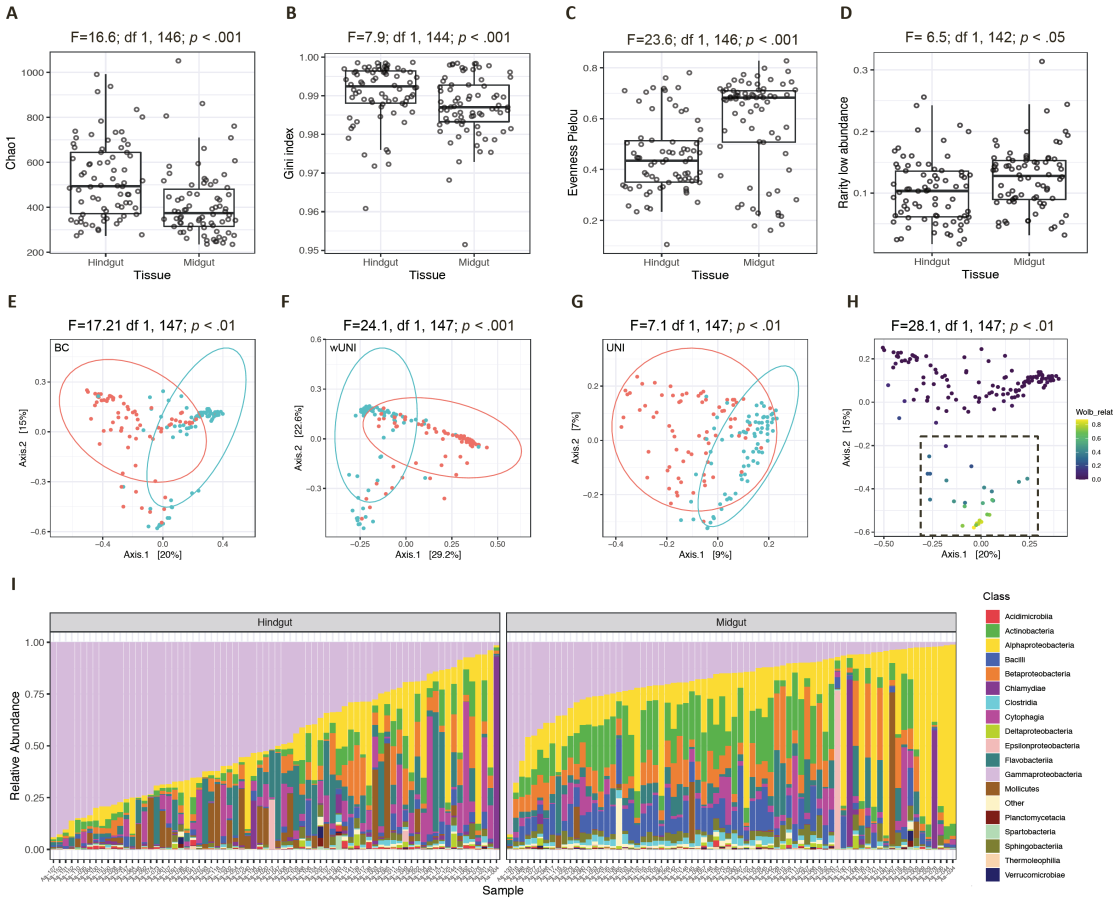

Suppl. Fig. 2. Microbiome diversity and composition between tissues

A-D. Alpha diversity estimates and **E-G** beta diversity estimates between tissues. In A-D, boxplots show the differences in Chao1 index (**A**), inequality (Gini index) (**B**), evenness (simpson evenness) (**C**), and rarity (of low abundance zOTUs) (**D**) between tissues. In **E-H** ordination plot of the principal coordinate analysis (PCoA) for individual

samples based on Bray-Curtis (BC) (**E**), weighted UniFrac (wUNI) (**F**) and unweighted Unifrac (UNI) (**G**) distances colored and grouped by tissue (hindgut in blue and midgut in red). Ellipses denote the 95% confidence interval. **H.** Ordination plot of the principal coordinate analysis (PCoA) for individual locations based on Bray Curtis (BC) distances colored by the relative abundance of *Wolbachia*. The dashed rectangle indicates the 30 individuals with a higher than the mean *Wolbachia* abundance that were considered the *Wolbachia* positive individuals. **I.** Barplots show relative abundance per individual sample and gut tissue of different bacterial taxa at the level of phylum (left) and class (right). **I.** Stacked bar plots show relative abundance per individual sample and tissue of different bacterial taxa at the level of class.

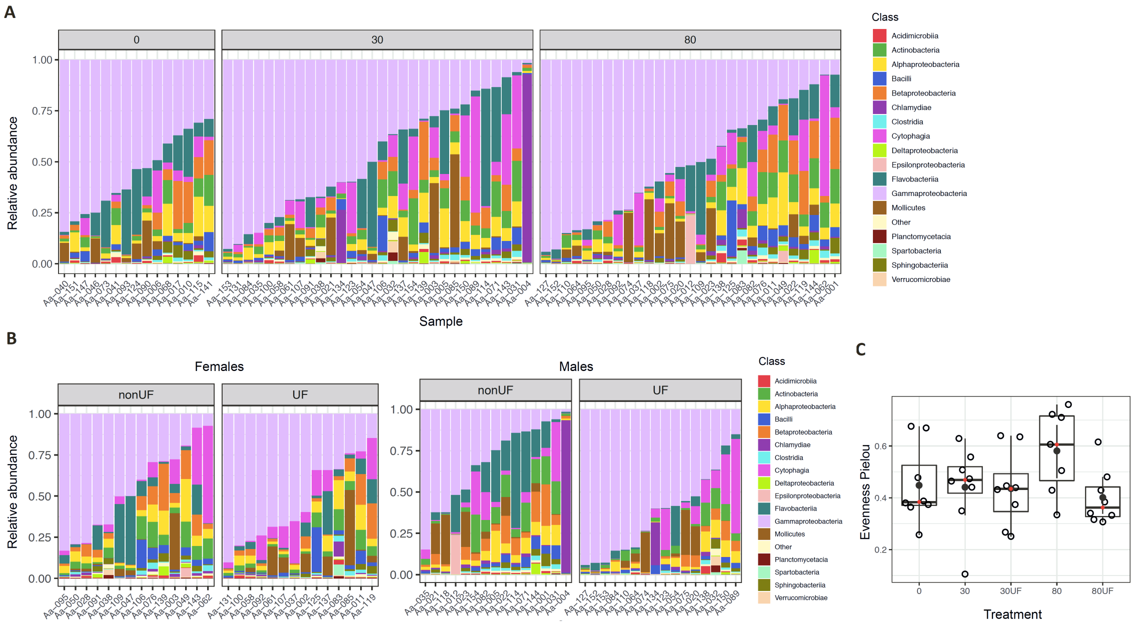

Suppl. Fig. 3. Microbiome diversity and composition between tissues

A. Stacked bar plots show relative abundance per individual sample and wastewater concentration (0%, 30%, and 80% WW) of different bacterial taxa at the level of class. **B.** Stacked bar plots show relative abundance per individual sample and ultrafiltration treatment (ultrafiltered: UF, non-ultrafiltered: non-UF) of different bacterial taxa at the level of class. **C.** Boxplot shows the differences in evenness (Pielou index) in females between treatments.
