# Supplementary material for: Effects of anthropogenic stress on hosts and their microbiomes: Treated wastewater alters performance and gut microbiome of a key detritivore (Asellus aquaticus)
Source: Evol Appl. 2023 Mar 30;16(4):824–48. doi: 10.1111/eva.13540 (PMC10130563; doi:10.1111/eva.13540)
Supplement: Supplementary file 3 — Annex S1. [file EVA-16-824-s003.pdf]

Annex 1. Detailed descriptions of methods for chemical analysis with HPLC-MS/MS

Before MP analysis, LC-vials of the samples were thawed and centrifuged. From each LC-vial, the top 1 mL was transferred to a new LC-vial. The samples were spiked with ISTD (Internal Standard, 20 µL, final conc. 200 ng L-1). The LC-MS-method used is described by Hagemann et al. (2020), with some modification. Briefly, direct injection of 100 µL per sample was performed on an Agilent 1290 Infinity LC System equipped with an Acquity UPLC HSS T3 (1.8 µm, 3.0x100 mm, Waters) column for chromatographic separation, coupled to a triple quadrupole MS (Agilent TQ6495C) for detection. The electrospray ionization was operated with a capillary voltage of 3500 V in positive and 3000 V in negative mode and a dynamic MRM with 650 ms cycle time. The LC System was operated at a flow rate of 0.5 mL min-1 with a gradient of 100% eluent A (nanopure water plus 0.1% formic acid) to 95% eluent B (methanol plus 0.1% formic acid) in 18.5 min. Hold for 3.5 min, go to 100% eluent A in 0.5 min and hold for 4.5 min.

The quantification of MPs was performed by using ISTD and a standard calibration curve between 0.5 – 7'500 ng L-1. The applied software was MassHunter Quantitative Analysis Version B.08.00 for QQQ (Agilent Technologies). Two transitions were analysed for quality control. The qualifier recovery was calculated as the ratio between the quantifier transition and qualifier transition with a tolerance between 80 – 120 %. For relative recovery calculation, four samples in total were spiked with a known concentration of the analysed compounds. The relative recovery was calculated with the concentration of the spiked sample minus the concentration of the unspiked sample, divided by the theoretical concentration spiked. The average was taken for each substance. The relative recoveries ranged from 91% to 130% for all substances and samples analysed quantitatively (Table S5). For substances without own ISTD, concentrations were corrected by the relative recovery. For calculation of the limit of quantification (LOQ), the lowest calibration standard found (with S/N 10:1) was divided by the matrix factor. The LOQs ranged between 5 to 223 ng L-1 (Table S5). The matrix factor was calculated with the area of the ISTD in the sample divided by the average of the areas of the ISTD in the calibration row for substances with an own ISTD. The average was taken for each substance. For substances without own ISTD, the area of an unspiked sample was substracted from the area of the corresponding spiked sample and the result was divided by the average of the areas of the calibration points with the same concentration. The average was taken for each substance. Five substances (Acesulfame, Chloridazone-methyl-desphenyl, Cyclamate, DEET, and Naproxen) were only analysed semi-quantitatively due to calibration- or peak-shape constraints.
